# Improved nucleic acid sensing, exemplified using pure target DNA sequences, by MO capture probes compared to DNA capture probes, as revealed from PO_2_^−^, PO_3_^−^ and nucleobase mass peaks

**DOI:** 10.1039/d6ra03286a

**Published:** 2026-09-17

**Authors:** Budhaditya Bhattacharya, Jayanta Kundu, Aratrika Ghosal, Asmita Paul, Md Qasim, Sirsha Dattasarma, Tanushree Mana, Subhra Sankar Dutta, Surajit Sinha, Sayan Dey, Swati Dasgupta, Rupa Mukhopadhyay

**Affiliations:** a School of Biological Sciences, Indian Association for the Cultivation of Science Kolkata-700032 West Bengal India bcrm@iacs.res.in +91 33 2473 2805 +91 33 2473 4971 ext. 1506; b School of Applied & Interdisciplinary Sciences, Indian Association for the Cultivation of Science Kolkata-700032 West Bengal India; c School of Chemical Sciences, Indian Association for the Cultivation of Science Kolkata-700032 West Bengal India; d CliniMed LifeSciences Stesalit Tower, E-2-3, GP Block, Sector V, Bidhannagar Kolkata-700091 West Bengal India

## Abstract

Detection of gene mutations is central to molecular diagnostics. The conventional detection methods that employ DNA capture probes are widely used, yet they may suffer from non-specific signals, difficulties in single nucleobase mismatch discrimination, and nuclease-induced degradation of the DNA capture probe. Herein, a strategy for detection of nucleic acid sequences using the nuclease-resistant morpholino oligonucleotide (MO) capture probe is reported. Since, spherical NPs offer increased surface area, thus increasing the total number of capture probe available for target recognition, MO probes were immobilized onto spherical NPs. Time-of-flight secondary ion mass spectrometry (TOF-SIMS), a sensitive surface analysis method, was employed to assess the status of target binding. Target binding was confirmed by recognizing the differences in chemical structures of MO and DNA, *i.e.*, high-intensity peaks for PO_2_^−^ and PO_3_^−^ were observed when fully complementary target DNA was introduced to the sensor surface, but not for the non-complementary DNA and MO-only surface. The MO-based detection offered significantly improved performance than the DNA-based system in discriminating complementary and non-complementary DNA sequences, including the sequences having the most prevalent driver mutations in exon 19 and exon 21, from non-small cell lung cancer (NSCLC) patients, with limit of detection of 1 pM, and machine learning validation. Since MO is devoid of PO_3_^−^, the MO capture probe-based detection of DNA sequences was unambiguous.

## Abstract 2

Unambiguous MO capture probe-based detection of nucleic acid sequences using PO_3_^−^ mass peaks.

## Graphical abstract

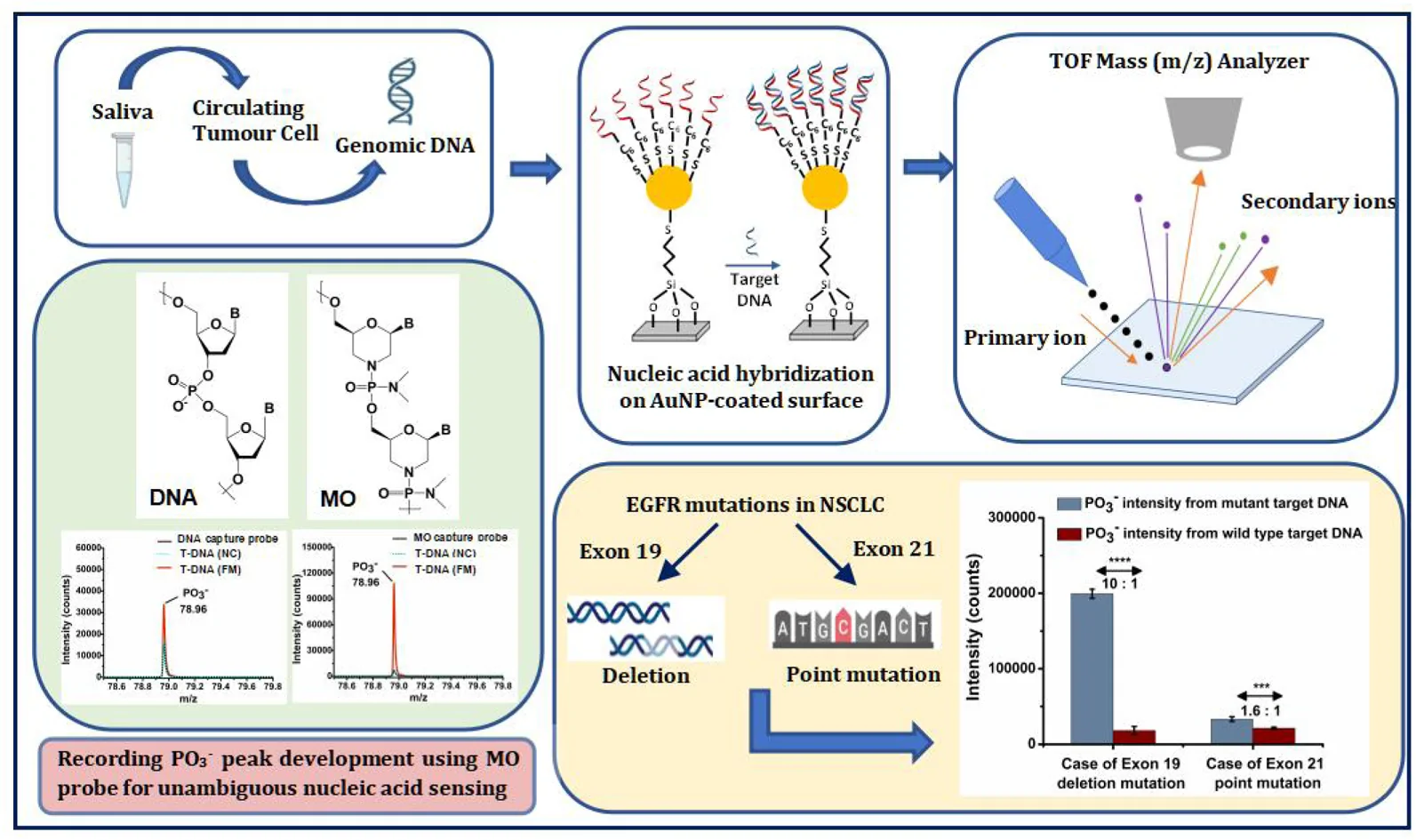

## Introduction

Nucleic acids are the main genetic elements in an organism, responsible for propagation of information to next generations and proper functioning of the biological systems. Therefore, alterations in the DNA/RNA sequences can lead to detrimental outcome. Different types of alterations can lead to various diseases like sickle cell anaemia, β-thalassemia, cardiovascular diseases, neurodegenerative diseases and cancer among many others.^1^ Identification of such altered genetic sequences is important for early diagnosis of the disease or its recurrence and also for the assessment of susceptibility to a genetic disease like cancer, Alzheimer's disease and tuberculosis. Furthermore, the detection of specific DNA/RNA sequences is important for identification of various micron/sub-micron level pathogenic organisms like *E. coli*, *Salmonella*, Ebola virus, Nipah virus *etc.* in environment, food and water.

**Fig. 1:**
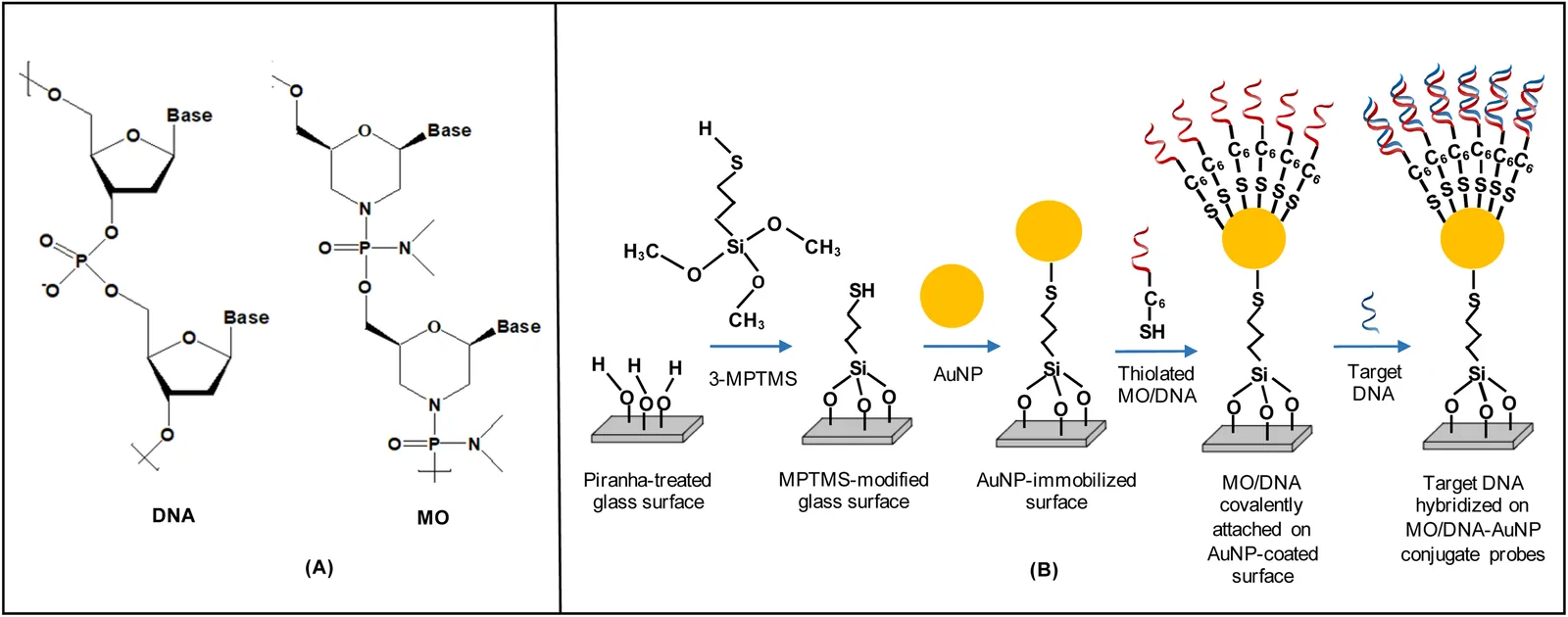
(A) Chemical structures of DNA (deoxyribonucleic acid) and MO (morpholino oligonucleotide). (B) A schematic diagram on fabrication of MO/DNA-AuNP conjugate-modified glass surface for on-surface detection of target DNA.

In recent years, a number of techniques have been employed for nucleic acid sensing,^2,3^ which mostly have utilized the DNA capture probes. Though the DNA-based sensors have shown considerable sensitivity, they also suffer from limitations like poor reproducibility, non-specific signal generation, instability of DNA capture probe *etc.* To overcome the limitations of the DNA-based biosensors, application of synthetic nucleic acids as an alternative to the DNA capture probe, such as, locked nucleic acid (LNA) and peptide nucleic acid (PNA) has been exemplified.^4–6^ A non-ionic alternative nucleic acid structure named morpholino oligonucleotide (MO)^7^ exhibits excellent specificity towards complementary nucleic acid sequences along with the properties like rigid backbone, resistance to nuclease/protease, low toxicity and high solubility in aqueous medium. Due to the distinct structural differences in the backbones of DNA and MO in terms of charge (Fig. 1A), *i.e.*, DNA being negatively charged and MO being non-ionic, MO-DNA duplex is also more stable than the DNA–DNA duplex. Previously, we have shown that surface-confined MO capture probe can be used to detect single nucleobase mismatches in nucleic acid sequences using atomic force microscopy (AFM)-based single molecule force spectroscopy approach (SMFS).^8^ Apart from this study, there are only a few instances where MO has been used as the capture probe for nucleic acid sensing.^9–11^

In the present study, MO capture probes have been anchored onto spherical gold nanoparticles, which are attached onto glass or silicon surface (Fig. 1B). The time-of-flight secondary ion mass spectrometry (TOF-SIMS), which is a surface-sensitive method for characterization of the top 2–10 Å (or the first two to three monolayers) of a solid surface at elemental/molecular level,^12,13^ was used for characterization of the sensor surface at each step of its fabrication, and finally for detection of target DNA upon hybridization with the surface-anchored MO capture probes. In SIMS, the surface of interest is bombarded with ions or clusters of ions (known as primary ions) that results in an energy-induced collision cascade confined to the top layers of the solid surface. This collision cascade results in the sputtering of ions, both positive and negative, known as secondary ions. These secondary ions are detected in the mass detector in time-of-flight manner, generating the mass spectra for the secondary ions. The mass resolution (>6000 m/Δ*m*) in SIMS is high enough so as to differentiate between species with very close masses. An added advantage of SIMS is that no fluorescent labelling is needed. Therefore, no extra step is involved for sample modification with a fluorescent probe. SIMS provides direct information, unlike in case of fluorescence-based detection, and is also devoid of the problems of false signals as sometimes encountered in fluorescence detection experiments. Herein, SIMS has been used to identify the target-specific binding of DNA sequences to the MO capture probes following Watson–Crick base pairing. SIMS has been used previously to detect the target DNA sequences using DNA capture probes^14,15^ and PNA capture probes.^16^ To our knowledge, this is the first report of a TOF-SIMS-based DNA sensing study using MO capture probes. Importantly, capacity of SIMS to discriminate single base mismatches is also reported for the first time. In addition, this is the first SIMS-based report for detection of clinically relevant samples (here, from non-small cell lung cancer patients) in PCR-independent manner. The limit of detection achieved (1 pM) is among the best reported for SIMS-based DNA sensing.

## Materials and methods

### Chemicals and bio-materials

Citrate-capped, spherical gold nanoparticles of diameter 10 nm were purchased from Sigma-Aldrich (Merck) (St. Louis, MO, USA) and the solution was diluted in autoclaved filtered Milli-Q water (resistivity 18.2 MΩ cm) for subsequent uses. The 12-mer and 15-mer 3′-thiolated MO sequences were synthesized using established protocols.^8,17^ MO being a highly water-soluble molecule, it was dissolved in Milli-Q water. Thiolated 12-mer single stranded DNA capture probes were procured from Alpha DNA (Montreal, Canada). The 12-mer fully complementary, 12-mer singly mismatched and 12-mer fully non-complementary target DNAs without any labelling were purchased from GCC Biotech (West Bengal, India). Microscope glass slides were used for preparation of the MO-modified surfaces. Acetone and propanol, which were used for surface cleaning, were procured from Sigma-Aldrich (Merck) (St. Louis, MO, USA). For buffer preparation, sodium chloride (NaCl), disodium hydrogen phosphate (Na_2_HPO_4_), sodium dihydrogen phosphate (NaH_2_PO_4_), tri-sodium citrate dihydrate (Na_3_C_6_H_5_O_7_·2H_2_O) were purchased from Sigma-Aldrich (Merck) (St. Louis, MO, USA). 98% H_2_SO_4_ and 30% H_2_O_2_ were purchased from Sigma-Aldrich (Merck) (St. Louis, MO, USA). 3-Mercaptopropyltrimethoxysilane (MPTMS) and 1,4-dithiothreitol (DTT) were purchased from Sigma-Aldrich (Merck) (St. Louis, MO, USA).

### Preparation of DNA capture probe solutions

Single-stranded DNA capture probes containing a hexyl thiol [–(CH_2_)_6_SH] group at the 5′-end (HS-DNA-1) (see Table 1) were dissolved in sodium citrate buffer (0.3 M trisodium citrate dihydrate, 3 M sodium chloride, pH 4.5). HS-DNA-1 was used as the capture probe against fully complementary target DNA as well as fully non-complementary target DNA. The concentrations of DNA solutions were measured by UV-visible spectrophotometry using a NanoDrop One^C^ spectrophotometer (Thermo Fisher Scientific, Waltham, MA, USA), for absorbance at 260 nm, and the molar extinction co-efficient (*ε*_260_) values for HS-DNA-1 as 1.23 × 10^5^ M^−1^ cm^−1^.

**Table 1:** The nucleic acid sequences used in SIMS experiments [FM: full match; SMM: single mismatch; NC: non-complementary; WT: wild type; Mut: mutant]

Capture probe/target sequence	Nucleic acid sequence
HS-MO-1	3′-HS-C_6_-CTA-TGT-CAG-CAC-5′
HS-DNA-1	5′-HS-C_6_-CTA-TGT-CAG-CAC-3′
T-DNA-1_FM_	5′-GAT-ACA-GTC-GTG-3′
T-DNA-2_FM_	5′-GTG-CTG-ACA-TAG-3′
T-DNA_NC_	5′-CGA-TAG-TCT-AGC-3′
T-DNA_SMM_	5′-GAT-ACA-GG̲C-GTG-3′
HS-MO_EXON 19_	3′-HS-C_3_-TAG-TTC-TGT-AGA-GGC-5′
T-DNA_EXON 19 (WT)_	5′-GAA-TTA-AGA-GAA-GCA-3′
T- DNA_EXON 19 (Mut)_	5′-ATC-AAG-ACA-TCT-CCG-3′
HS-MO_EXON 21_	3′-HS-C_3_-AAA-CCC-GCC-CGG-TTT-5′
T-DNA_EXON 21 (WT)_	5′-TTT-GGG-CTG-GCC-AAA-3′
T- DNA_EXON 21 (Mut)_	5′-TTT-GGG-CG̲G-GCC-AAA-3′

### Preparation of MO capture probe solutions

The 12-mer and 15-mer thiolated single-stranded MO sequences (HS-MO-1, HS-MO_EXON 19_, HS-MO_EXON 21_) (Table 1) were synthesized using the established protocol^8,17^ and purified using HPLC. The MO solutions were prepared in Milli-Q water (resistivity 18.2 MΩ cm). For subsequent immobilization steps too, Milli-Q water was used. The concentrations of MO solutions were determined by UV-visible spectrophotometry using a NanoDrop One^C^ instrument *via* monitoring absorbance at 260 nm and using the molar extinction co-efficient (*ε*_260_) of HS-MO-1 as 1.22 × 10^5^ M^−1^ cm^−1^, HS-MO_EXON 19_ as 1.71 × 10^5^ M^−1^ cm^−1^ and HS-MO_EXON 21_ as 1.63 × 10^5^ M^−1^ cm^−1^.

### Preparation of target DNA solutions

For hybridization with the surface-attached HS-MO-1 probes, T-DNA-1_FM_ (Table 1) solution was prepared in 20 mM sodium phosphate, 20 mM sodium chloride, pH 7.0 buffer prior to hybridization reaction. The concentration was measured by UV-visible spectrophotometry using a NanoDrop One^C^ instrument. The T-DNA-1_FM_ sequence was fully complementary to the MO-1 capture probe. T-DNA_SMM_ (Table 1) was used as the singly mismatched target DNA against MO capture probe. The sequence contained one nucleobase mismatch (T > G) located in the central position. T-DNA_SMM_ solution was prepared in 20 mM sodium phosphate, 20 mM sodium chloride, pH 7.0 buffer.

When hybridization reaction was performed against DNA capture probe (HS-DNA-1), T-DNA-2_FM_ (Table 1) solution was used which had the same nucleobase sequence as T-DNA-1_FM_ differing only in the 5′–3′ orientation/direction as the MO and DNA capture probes differed in the orientation of thiol attachment (3′ in MO and 5′ in DNA capture probe), and target DNA T-DNA-2_FM_ solution was prepared in 20 mM sodium phosphate, 100 mM sodium chloride, pH 7.0 buffer. The concentration of the target DNA was verified by UV-visible spectrophotometry using a NanoDropOne^C^ instrument. The T-DNA-2_FM_ was fully complementary to HS-DNA-1 capture probe. The non-complementary target DNA (T-DNA_NC_) (Table 1), which was non-complementary to both the capture probes, *i.e.*, HS-MO-1 and HS-DNA-1, was prepared in buffers 20 mM sodium phosphate, 20 mM sodium chloride, pH 7.0 for experiment with HS-MO-1 and 20 mM sodium phosphate, 100 mM sodium chloride, pH 7.0 for experiment with HS-DNA-1.

When hybridization between two ssDNA strands occurs, the negatively charged surface-attached DNA capture probe strand repels the negatively charged target DNA strand. To neutralize the negative charges, positively charged ions like Na^+^ or Mg^2+^ are added in the hybridization reaction solution. The target DNA, T-DNA-1_FM_ was fully complementary to MO capture probe and this target DNA solution was prepared in phosphate buffer, containing 20 mM sodium chloride, as MO is non-ionic in nature, thus there will be no need of high salt concentration to shield any negative charge around the capture probe strands. When thiolated DNA was the capture probe, then T-DNA-2_FM_ was used which was prepared in phosphate buffer containing 100 mM sodium chloride. In this case, high salt concentration was required to neutralize/reduce the negative charge of the DNA strands, as otherwise, the negatively charged DNA capture probe would repel the negatively charged target DNA.

In case of target DNA sequences having EGFR gene (wild type and mutated) from non-small cell lung cancer (NSCLC) patients, the DNA sequences were obtained from the circulating tumour cells (CTCs) in saliva of the patients, and healthy individuals (wild type, as control). Two mutations were selected, one is the exon 19 deletion mutation and the other one is the exon 21 point mutation, as they are the most prevalent mutations in case of NSCLC.^18^ T-DNA_EXON 19 (Mut)_ (Table 1) having exon 19 deletion mutation was taken in 20 mM sodium phosphate buffer, 20 mM sodium chloride, pH 7.0 buffer for hybridization with corresponding MO capture probe (HS-MO_EXON 19_). T-DNA_EXON 19 (Mut)_ sequence was fully complementary to HS-MO_EXON 19_. The wild type exon 19 DNA sequence, T-DNA_EXON 19 (WT)_ was non-complementary to HS-MO_EXON 19_. The exon 21 point mutated (T > G) target DNA sequence T-DNA_EXON 21 (Mut)_ was also prepared in 20 mM sodium phosphate, 20 mM sodium chloride, pH 7.0 buffer prior to exposure to surface attached-MO capture probe HS-MO_EXON 21_. T-DNA_EXON 21 (Mut)_ sequence was fully complementary to HS-MO_EXON 21_. The wild type DNA, T-DNA_EXON 21 (WT)_, isolated from healthy individuals, contained a single base mismatch in the central position to the HS-MO_EXON 21_. In all cases of sequences having exon 19 and 21 mutations, and corresponding wild type sequences, they were obtained by first fragmenting the whole genome from CTCs, then capturing the sequences of interest by using respective complementary locked nucleic acid (LNA) (Eurogentec, Belgium) probes anchored onto gold surface, and finally melting the LNA-DNA duplex at 70 °C to retrieve the DNA sequences in solution. The MO capture probes were designed in a way so that the mutated T-DNA sequences were fully complementary and the strongest SIMS signal could be observed upon hybridization with the respective mutated sequences.

### Characterization of gold nanoparticles

The colloidal solution of spherical gold nanoparticles was prepared in Milli-Q water. Before using the nanoparticles for biosensor surface fabrication, they were characterized using UV-visible spectrophotometry (Cary 50 spectrophotometer, Agilent Technologies, Santa Clara, CA, USA), dynamic light scattering (DLS) (Malvern Panalytical, Worcestershire, UK) and high-resolution transmission electron microscopy (HR-TEM) (JEOL 100CX) (JEOL Ltd., Tokyo, Japan).

### Fabrication of MO/DNA-AuNP-conjugate modified surface

To prepare the sensing surface for detection of nucleic acids, the microscope glass slides, which are readily available and cost-effective, were cut into 10 × 10 mm^2^ sized pieces. The glass pieces were then washed by a three-step cleaning process employing acetone, 2-propanol and filtered autoclaved Milli-Q water by bath sonication in each of the solutions for 10 min.^19^ The glass pieces were then immersed in freshly-prepared piranha solution [H_2_SO_4_ : H_2_O_2_ = 2 : 1 (v/v)] for 1 h at room temperature. Apart from cleaning the surface from organic contaminants, the piranha treatment exposed the hydroxyl (–OH) groups on glass surface, which is a prerequisite for silanization of the surface. Then the surfaces were washed thoroughly in filtered autoclaved Milli-Q water by bath sonication for 10 min and dried under soft nitrogen jet. The piranha-treated glass surfaces were then immersed in 100 mM 3-MPTMS solution prepared in 2-propanol in presence of 50 mM DTT and incubated for 12 h at room temperature. After the incubation period was over, the surfaces were sonicated in 2-propanol, ethanol and Milli-Q water for 5 min in each of the solutions and finally dried with soft nitrogen jet. The binding of the MPTMS molecules on glass surface occurred through the condensation reaction between the methoxy (–OCH_3_) groups of MPTMS and the exposed hydroxyl (–OH) groups on the glass surface (Fig. 1B). In order to prepare a thin film of gold nanoparticles, 5 nM colloidal gold solution was deposited onto the MPTMS-modified surface and kept for 8 h at room temperature. The surface was rinsed with Milli-Q water to remove the unbound AuNPs from surface. On the AuNP-coated surface, the thiolated single-stranded MO/DNA was immobilized by depositing a 30 µL droplet of the respective MO/DNA solution. For the HS-DNA-1 capture probes, 0.5 µM concentration was used. After 12 h incubation, the DNA-coated surface was washed with sodium citrate buffer, and finally with filtered autoclaved Milli-Q water. For the MO-1 capture probes, 0.5 µM concentration was used. After 10 h incubation, the MO-modified surface was washed with filtered autoclaved Milli-Q water. After washing, the surfaces were dried with nitrogen jet and used for detection of the target DNA sequences. The immobilization steps are shown in Fig. 1B.

### Surface imaging by atomic force microscopy (AFM)

High-resolution AFM images were obtained from AuNP-coated glass surface to assess the surface coverage after immobilization of nanoparticles on MPTMS-modified surface. The imaging was performed using an MFP-3D AFM equipment (Asylum Research, Santa Barbara, CA, USA) in ambient condition at room temperature (24 ± 0.5 °C) and 45–50% humidity with a 90 µm scanner. Imaging was carried out in the non-contact mode to minimize sample damage. The AFM tip (NSC 35 AlBS, µMasch, Estonia) used for the experiment had a spring constant of 1.7–14 N m^−1^ and frequency range 95–205 kHz. The AFM tip was cleaned using a UV-ozone cleaner (BioForce Nanosciences, Ames, IA, USA) before imaging.

### Detection of target DNA sequences *via* hybridization on MO/DNA-AuNP conjugates

The MO or DNA-modified surface was first washed with the buffer in which target DNA solutions were prepared (20 mM sodium phosphate, 20 mM sodium chloride, pH 7.0 for MO-DNA hybridization case, and 20 mM sodium phosphate, 100 mM sodium chloride, pH 7.0 for DNA–DNA hybridization case) and dried with nitrogen jet. On this surface, 30 µL droplet of target DNA solution (1 µM concentration or less) was deposited and kept inside a humidity chamber for 2 h. After the incubation period was over, the surface was washed with the respective phosphate buffer and finally with filtered autoclaved Milli-Q water and dried under soft nitrogen jet. For addressing the case of non-complementary situation (control experiment), the sequence T-DNA_NC_ was deposited onto DNA/MO capture probe-modified surfaces under similar experimental condition.

### Data acquisition and analysis by secondary ion mass spectrometry (SIMS)

In the present work, a TOF-SIMS 5 instrument (IONTOF GmBH, Münster, Germany) was used for mass spectrometry data acquisition with a reflectron type mass detector in static SIMS mode (ion dose density <10^12^ ions per cm^2^) using Bi_3_^+^ as the primary ion source over an area of 256 × 256 µm^2^ with the data acquisition time being 5 min for negative ions and 3 min for positive ions. As SIMS is a semi-quantitative technique, the mass spectra from control surfaces (*i.e.*, bare glass surface, MPTMS-modified surface, AuNP-coated surface) were acquired for comparison. Data collection was done from three different points of a surface and averaged. The experiments were repeated three to five times, and using different batches of samples. The ‘*n*’ value has been 3–5 considering all sorts of SIMS data acquisition. The error bars have been calculated for standard deviation. Data analysis, including peak identification and statistical validation, required approximately 10–15 min per sample.

### Statistical analysis

TOF-SIMS data are presented as mean ± standard deviation (*n* ≥ 3). Welch's *t*-test (unpaired, two-tailed) was used to determine the statistical significance of differences between experimental groups, with a *p*-value < 0.05 considered significant. The test was used to validate the specificity of DNA hybridization, to compare the capture efficiency of MO and DNA probes, and to confirm the discrimination of single-base mutations in patient-derived samples. For the concentration-dependence study, pairwise *t*-test and linear regression were employed. All statistical calculations were performed using GraphPad Prism 8.0.

### Non-negative matrix factorization (NMF) of TOF-SIMS spectra

Unsupervised machine learning *via* non-negative matrix factorization (NMF) was applied to the full-range negative-ion TOF-SIMS spectra (*m*/*z* 1–1000) to identify spectral components associated with experimental conditions. Spectra from three groups, MO only, MO with non-complementary DNA, and MO with fully complementary DNA, were normalized to total ion count and aligned by interpolating each spectrum onto the native *m*/*z* grid of a reference spectrum. NMF was implemented using the sklearn.decomposition. NMF module in Python (scikit-learn library). Factorization rank was optimized by evaluating reconstruction error, consensus cophenetic coefficient, and silhouette score across ranks *k* = 2–9. The resulting components represent non-negative basis spectra, and their corresponding activation weights quantify the contribution of each component to each individual spectrum.

## Results and discussion

### Characterization of gold nanoparticles

The purity and concentration of the colloidal gold nanoparticle solution was checked using UV-visible spectrophotometry. In all cases, a localized surface plasmon resonance (LSPR) absorption peak at 520 nm, which is the characteristic absorption peak for the AuNPs of 10 nm diameter,^20^ was observed (Fig. S1). The size and morphology of the AuNPs were checked using high-resolution transmission electron microscopy (HR-TEM). The atomic resolution HR-TEM images (Fig. S2) showed the (111) faces having 3-fold hollow sites on gold nanoparticle surfaces, which are the most preferred sites for attachment of the thiol (–SH) groups onto AuNP. Dynamic light scattering (DLS) study revealed the average hydrodynamic diameter to be 13.88 nm using Gaussian function (Fig. S3), which is slightly larger than the particle core as the surrounding stabilizer molecules are also included as part of the sphere, and the polydispersity index (PDI) was found to be 0.2, indicating an acceptable size distribution for commercial 10 nm AuNPs. All these data collectively suggest that the AuNPs in use were spherical in shape, their size distribution was uniform, they were well dispersed in solution and contained the (111) hollow sites for attachment of the –SH groups of the organosilanes and the thiolated oligonucleotides (Fig. 1B).

### Evidence of surface modification and presence of MO/DNA capture probes on surface

**Fig. 2:**
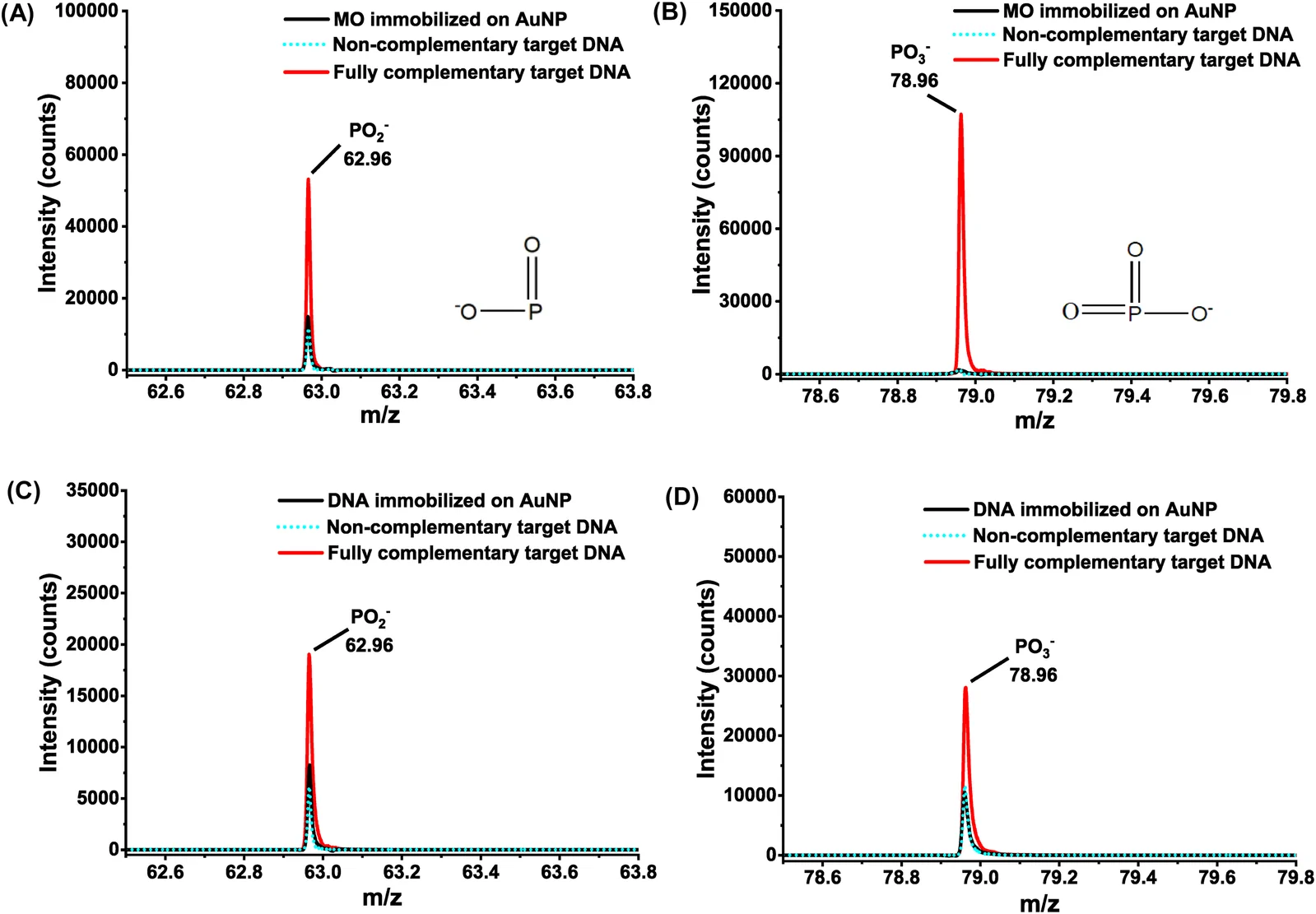
TOF-SIMS peaks (*m*/*z*) in negative ion mode presented for (A) PO_2_^−^ peak at 62.96 for the case of MO on AuNP surface, overlaid with the case of MO exposed to fully non-complementary DNA, and with the case of MO exposed to fully complementary DNA sequences; (B) PO_3_^−^ peak at 78.96 for the case of MO on AuNP surface, overlaid with the case of MO exposed to fully non-complementary DNA, and with the case of MO exposed to fully complementary DNA sequences. The high intensity peaks at 62.96 and 78.96 were observed only when fully complementary target DNA was hybridized with the MO capture probes. (C) and (D) represent corresponding peaks (*m*/*z*) for PO_2_^−^ and PO_3_^−^, for the case of DNA capture probe. Chemical structures of PO_2_^−^ and PO_3_^−^ are shown in the insets of (A) and (B), respectively.

**Fig. 3:**
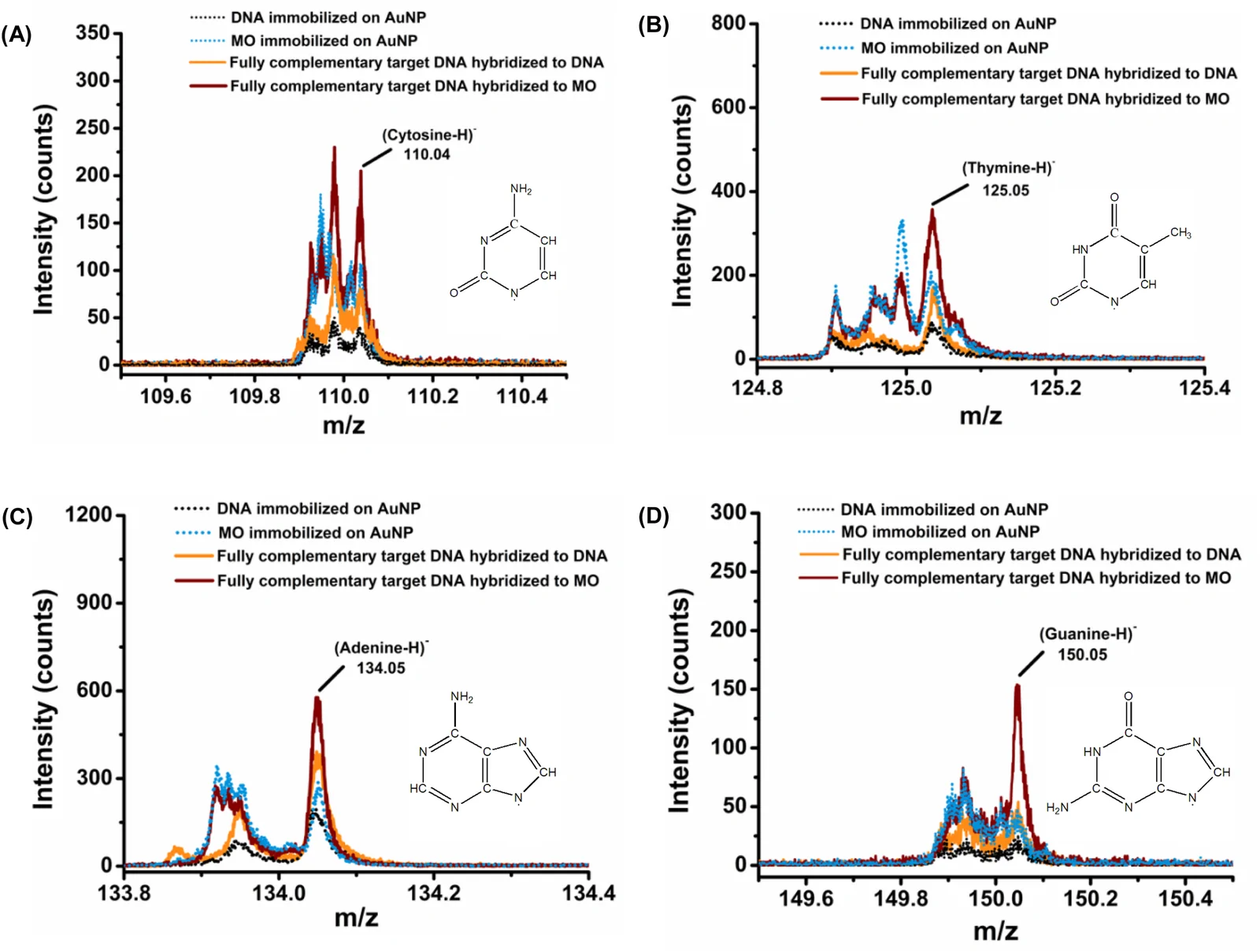
TOF-SIMS negative ion mass peaks (*m*/*z*) for molecular ions of (A) (cytosine-H)^−^ at 110.04, (B) (thymine-H)^−^ at 125.05, (C) (adenine-H)^−^ at 134.05 and (D) (guanine-H)^−^ at 150.05 for DNA capture probe on AuNP-coated surface, MO-capture probe on AuNP-coated surface and fully complementary target DNAs hybridized with corresponding DNA and MO capture probes. The chemical structure is shown for each nucleobase in the inset of each figure.

Effective immobilization of gold nanoparticles onto the MPTMS-modified surface *via* formation of Au–S covalent linkages, where the Au–S linkages offer stronger interaction than electrostatic interaction as in case of attachment of AuNPs through APTES treatment,^21,22^ is indicated in Fig. S4 and Table S1. The SIMS full-range spectra revealed the presence of gold-specific peaks (for Au^−^, Au_2_^−^, Au_3_^−^, Au_4_^−^*etc.*) (Fig. S4 and Table S1) confirming immobilization of AuNPs on glass surface. The surface morphological changes of the bare glass surface after modification with AuNPs have been revealed by AFM imaging (inset of Fig. S4), where the height difference between before and after AuNP coating is approximately 1.04 nm. The spatial distribution of AuNPs could be determined from SIMS imaging (Fig. S5). In addition to the gold ions, the presence of various gold–sulphur adducts were found, *e.g.*, AuS^−^, Au_3_S^−^*etc.* (Fig. S4 and Table S1). These results indicate that the gold nanoparticles were not merely physisorbed on the MPTMS-coated surface but they were chemisorbed *via* covalent bond formation with the thiol (–SH) groups of MPTMS. After AuNP immobilization, the glass chip was further modified with the MO or DNA capture probes. Both the capture probes were thiolated at one end (3′ end for MO capture probe and 5′ end for DNA capture probe) for facilitating the attachment of the capture probe oligonucleotides onto the AuNP-modified surface through thiol-AuNP interaction. Immobilization of the capture probes on AuNP-coated surface was detected using SIMS as evident from the characteristic peaks for nucleic acids (Fig. 2, 3 and Tables S2, S3). While comparing with the AuNP-coated surface, it was found that a strong peak appeared at *m*/*z* 62.96 for the MO/DNA-modified surface that accounted for PO_2_^−^ (Fig. 2A, C and S6). In order to assess any influence of the underlying substrate on the oligonucleotide-specific signals, the spectra obtained from bare substrate, MPTMS-modified substrate, AuNP-modified substrate, and capture probe-modified substrate (Fig. S6), were carefully observed and any matrix effect could be ruled out. From the structures of MO and DNA, it is evident that PO_2_^−^ ions can be generated from their backbone which is constituted by phosphorodiamidate linkages in case of MO and phosphodiester linkages in case of DNA. Apart from the PO_2_^−^ ion, a peak for PO_3_^−^ ion at *m*/*z* 78.96 was also observed in case of DNA capture probes (Fig. 2D), whereas this mass peak was absent in case of the MO-modified surface (Fig. S6D). The structural difference between MO and DNA backbones justifies the finding that a distinct and strong peak for PO_3_^−^ ion could not be observed in case of MO, as DNA backbone has 4 oxygens attached to phosphorus, whereas in the MO backbone, there are only two oxygens attached to phosphorus. To rationalize this observation at the molecular level, the SIMS fragmentation pathways of the two backbones must be considered. In DNA, the phosphodiester linkage contains four oxygen atoms bonded to phosphorus. Upon energetic ion bombardment, cleavage of the P–O bonds can generate PO_2_^−^ (*m*/*z* 62.96) as well as PO_3_^−^ (*m*/*z* 78.96), as the latter retains three oxygen atoms from the phosphate moiety. In contrast, the phosphorodiamidate linkage of MO contains only two oxygen atoms bonded to phosphorus, with the remaining two valences occupied by nitrogen atoms. Fragmentation of this structure can produce PO_2_^−^ but cannot yield PO_3_^−^, as the required third oxygen atom is simply not present in the local chemical environment. This structural constraint is experimentally confirmed by the complete absence of the PO_3_^−^ peak in case of MO-only surfaces (Fig. S6D). Therefore, in MO-based detection, the appearance of a PO_3_^−^ signal at *m*/*z* 78.96 provides unambiguous chemical evidence of successful target DNA hybridization, as this ion can only originate from the captured DNA's phosphodiester backbone.

The observation of the molecular ion peaks characteristic of all the four nucleobases further extended confirmatory evidence for successful immobilization of the MO/DNA capture probes (Fig. 3). The molecular ion peaks in the negative ion spectra were found in the form (base-H)^−^, thus for cytosine the mass peak was at *m*/*z* 110.04, for thymine the peak was found at 125.05, for adenine at 134.05 and for guanine the peak was found at 150.05. These peaks were not found in case of the control surfaces (*i.e.*, bare glass, MPTMS-modified surface and AuNP-coated surface).

An idea about loading efficiency of MO and DNA capture probes could be elicited from the respective peak intensity values for MO-only and DNA-only cases. The PO_2_^−^ peak intensity ratio (MO : DNA) is found to be 2.2 : 1, for (cytosine-H)^−^ peak intensity the ratio is 3.2 : 1, for (thymine-H)^−^, it is 2.7 : 1, for (adenine-H)^−^, it is 1.5 : 1, and for (guanine-H)^−^, it is 1.9 : 1. Therefore, the loading efficiency of MO capture probe is found to be considerably higher (about twice or thrice) compared to that of DNA capture probe. The higher immobilization density of MO capture probes compared to DNA probes (evidenced by 2–3 × greater PO_2_^−^ and nucleobase peak intensities, see Fig. 2A, C and 3) can be explained at the molecular level by two primary factors. First, the neutral morpholino backbone eliminates electrostatic repulsion between neighboring probes on the AuNP surface, whereas DNA's negatively charged phosphates cause mutual repulsion that limits packing. Second, MO's rigid six-membered ring backbone promotes an upright orientation, thereby maximizing packing and surface coverage, while DNA's flexibility leads to a less organized layer, reducing effective packing density. Additionally, the lower aqueous solubility of MO compared to DNA suggests reduced hydration, which may further contribute to minimizing steric hindrance and the effective hydrodynamic volume of each probe.^23^ Collectively, these factors can promote higher surface loading of MO.

### Hybridization of target DNA and its detection by TOF-SIMS

In the previous section, it has been shown that the MO capture probes were immobilized on the AuNP-coated glass surface as proven from the observation of the mass peaks corresponding to PO_2_^−^, (cytosine-H)^−^, (thymine-H)^−^, (adenine-H)^−^ and (guanine-H)^−^ at *m*/*z* 62.96, 110.04, 125.05, 134.05 and 150.05, respectively (Tables S2, S3 and Fig. 2, 3). After successful attachment of the MO capture probes on surface, target DNA solution (fully complementary or fully non-complementary or singly non-complementary) was applied onto the MO-coated surface.

A low salt concentration (20 mM sodium chloride) was used in hybridization buffer, as MO strands are non-ionic in nature. Thus, there would be no inter-strand repulsion between the MO capture probes and the target DNA strands, and high concentration of Na^+^ ions was not necessary to shield the anionic backbone as needed in case of detection of target DNA strands using DNA capture probes. As a control experiment, fully non-complementary DNA sequence was added to the MO-coated surface under the same experimental condition. After incubation and subsequent washing, the TOF-SIMS experiment was performed for the fully complementary target DNA-treated surface, non-complementary DNA-exposed surface and a reference MO-coated surface, all the samples placed in the SIMS chamber in one experiment for the purpose of comparison. The mass spectra, in negative ion mode, revealed that hybridization occurred between the surface-confined MO capture probes and the target DNA strands, as the intensity of PO_2_^−^ and PO_3_^−^ and the nucleobase peaks increased after hybridization (Fig. 2 and 3). It has been mentioned before that the mass peak for only PO_2_^−^ is significantly observed but not the PO_3_^−^ in case of MO, whereas in case of DNA, mass peaks for both PO_2_^−^ and PO_3_^−^ ions are observed in abundance. These observations were exploited to differentiate between single stranded MO, attached onto the AuNP-surface, and MO-DNA duplex formed due to hybridization on the surface. In Fig. 2B, it is shown that a high-intensity peak appeared at *m*/*z* 78.96 when fully complementary target DNA was hybridized with the MO capture probe, while there was no such intense peak when non-complementary target DNA was added and also when there was no target DNA at all (*i.e.*, for MO-only surface). Detection of target DNA hybridization was also indicated in the increase in intensity of the PO_2_^−^ peak at *m*/*z* 62.96 (Fig. 2A) when the MO-only surface and target DNA-exposed surfaces were compared. The peak intensity increased only for the fully-complementary target DNA. For the non-complementary target DNA, the intensity was same as that in case of the MO-coated surface. Thus, the mass peaks for both PO_2_^−^ and PO_3_^−^ ions could be discriminated between a fully complementary target DNA and a fully non-complementary target DNA – the discrimination ratio between fully complementary target and fully non-complementary target being 13.5 using PO_3_^−^ and 4.5 using PO_2_^−^ peak intensities. Thus, it is concluded that PO_3_^−^ ion is more suitable for identifying occurrence of hybridization of target DNA with its complementary surface-anchored MO capture probe.

For the DNA capture probe-modified surface, the target DNA solutions were prepared in 20 mM sodium phosphate, 100 mM sodium chloride, pH 7.0 buffer and the same buffer was used for hybridization between the target DNA strands and the DNA capture probes. In this case, higher salt concentration was used than the case of MO-DNA hybridization because of the anionic nature of the DNA capture probes that needed to be shielded with counter-ions (Na^+^) to minimise the repulsion between target DNA strands and DNA capture probes. Fully complementary target DNA-hybridized surface, fully non-complementary target DNA-exposed surface and DNA capture probe-modified surfaces were placed in the SIMS chamber and studied in a single experiment from the clearly distinguishable mass peaks for PO_2_^−^ and PO_3_^−^ ions. Intensity of both the peaks increased significantly after hybridization with target DNA (Fig. 2C and D). For the non-complementary DNA-exposed surface, the intensities for these peaks remained almost the same as that of the DNA capture probe-coated surface with no target DNA added thus discriminating effectively between fully complementary and fully non-complementary target DNA sequences.

To statistically validate the significance of the intensity differences, a Welch's *t*-test was performed. The analysis confirmed that the increase in peak intensity after hybridization with the fully complementary target DNA was statistically highly significant (*p* < 0.01) for both PO_2_^−^ and PO_3_^−^ ions when compared to the initial MO-coated surface and the surface exposed to the fully non-complementary DNA (Fig. S7A, B and Table S2). These robust *p*-values demonstrate that the dramatic increase in signal is a direct and specific result of successful DNA hybridization and is not due to random variation or non-specific binding. The specificity of the PO_2_^−^ and PO_3_^−^ ions as definitive markers for DNA hybridization was further corroborated by an unsupervised machine learning analysis of the full spectral data set. Non-negative matrix factorization (NMF) independently identified a spectral component dominated by PO_3_^−^ and PO_2_^−^, whose activation was exclusively and strongly elevated in spectra from surfaces hybridized with fully complementary target DNA (see SI, Section C: “Machine Learning-Based Spectral Fingerprint Analysis for DNA Hybridization”). This data-driven approach confirms that these phosphate fragments constitute a robust, multivariate fingerprint for successful duplex formation, reinforcing the conclusions drawn from direct peak intensity analysis. Statistical analysis using Welch's *t*-test was similarly performed on the data obtained from the DNA capture probe-functionalized surfaces (Fig. 2C and D). The results confirm that the peak intensities for both PO_2_^−^ and PO_3_^−^ ions after hybridization with the fully complementary target DNA are statistically significantly different from both the DNA-only surface and the surface exposed to the fully non-complementary DNA (*p* < 0.05; Fig. S7C, D and Table S2). This validates the specific detection of the target sequence using the DNA capture probe. Furthermore, the increase in intensity of the nucleobase-specific peaks (cytosine-H)^−^, (thymine-H)^−^, (adenine-H)^−^, (guanine-H)^−^ following hybridization with MO-capture probe, as visually observed in Fig. 3, was also confirmed to be statistically significant by quantitative analysis (Fig. S8). A significant increase in the intensities of the nucleobase peaks was also statistically confirmed for the DNA capture probe upon hybridization (Fig. S8).

**Fig. 4:**
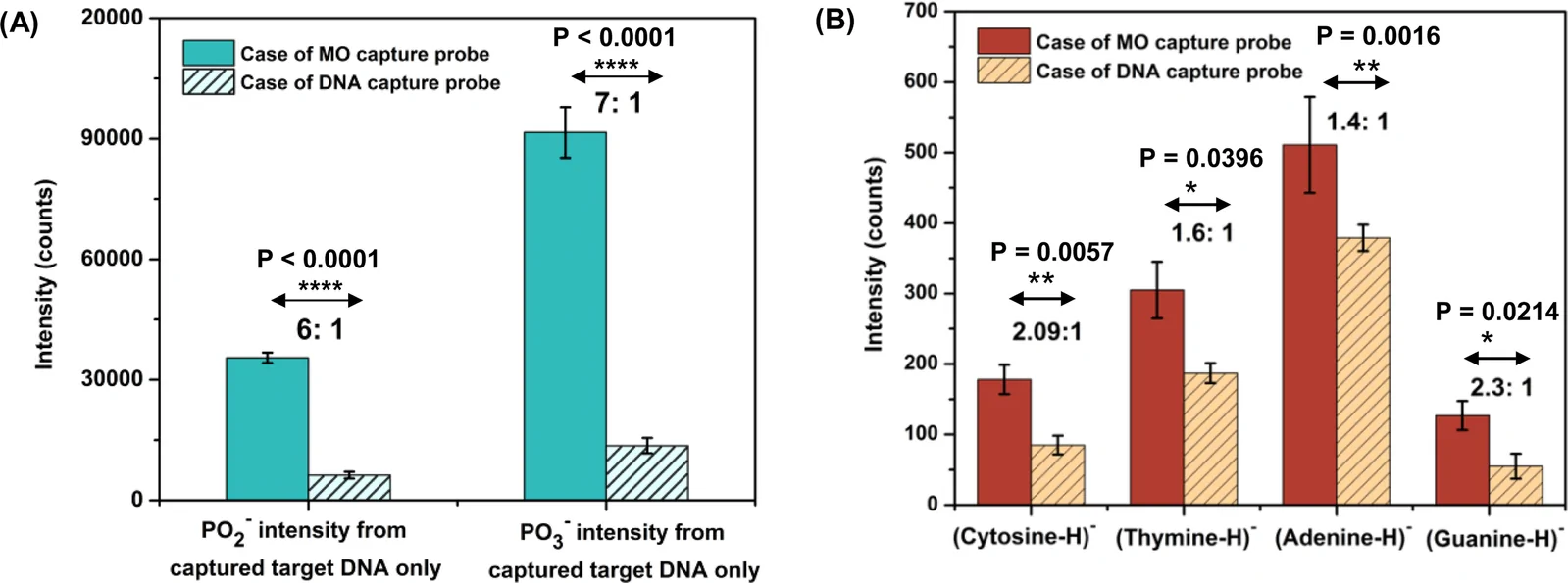
Bar diagrams (A) for comparing PO_2_^−^ and PO_3_^−^ intensity values at *m*/*z* 62.96 and 78.96, respectively, derived exclusively from fully complementary target DNAs captured by MO and DNA capture probes. The PO_2_^−^ intensity ratio 6 : 1 and the PO_3_^−^ intensity ratio 7 : 1 indicate significantly improved target DNA capturing ability of the MO capture probes compared to the DNA capture probes, a difference that is statistically highly significant (*p* < 0.0001, Welch's *t*-test); (B) for comparing the intensity values of molecular ions of nucleobases (cytosine-H)^−^, (thymine-H)^−^, (adenine-H)^−^ and (guanine-H)^−^ in negative ion mode at *m*/*z* 110.04, 125.05, 134.05 and 150.05, respectively, as observed for fully complementary target DNAs captured by MO and DNA capture probes. The intensity ratios of nucleobases for the case of MO-based detection compared to the case of DNA-based detection ranges from 1.4 to 2.3 showing superior performance of MO capture probe than its DNA counterpart. The signal intensities for all four nucleobases were significantly higher for the case of MO capture probe compared to the case of DNA capture probe (Welch's *t*-test, *p* < 0.05).

The peak intensity values for PO_2_^−^ and PO_3_^−^ ions for MO/DNA capture probe-coated surface, MO/DNA capture probe-coated surface after exposure to fully non-complementary target DNA and MO/DNA capture probe-coated surface after hybridization with fully complementary target DNA have been listed in Table S2. The intensities for PO_2_^−^ and PO_3_^−^, which act as indicators for target DNA detection, as derived exclusively from the captured fully complementary target DNAs (Table S4), are compared for both the cases of MO and DNA capture probes (Fig. 4A). It is evident from 6 times stronger PO_2_^−^ intensity and 7 times stronger PO_3_^−^ intensity for MO-based detection compared to DNA-based detection that MO capture probe is significantly more efficient capture probe than DNA capture probe. This superior performance was confirmed by Welch's *t*-test, which showed the difference in intensity between the cases for MO and DNA capture probes to be statistically highly significant (*p* < 0.0001). Since MO is non-ionic in nature, it does not repel the negatively charged single stranded target DNA molecules unlike the negatively charged DNA capture probe. Thus, greater number of target DNAs are hybridized with MO capture probes than with the DNA capture probes leading to substantially higher intensity of the PO_2_^−^ and PO_3_^−^ peaks arising from captured target DNA in case of MO probes. Moreover, since clear hybridization-specific signal can be obtained from the PO_3_^−^ peak when MO capture probe is used, where PO_3_^−^ intensity was totally coming from target DNA, this is another reason why MO-based DNA detection is better than DNA-based DNA detection. It is also proposed that PO_3_^−^ is the most suitable ion species for sequence-specific detection of target DNA using the SIMS-based method since the PO_3_^−^ ions are exclusively generated from the hybridized DNA molecules. The discrimination ratio between fully complementary target DNA and fully non-complementary DNA is found to be ∼1.6 for both PO_2_^−^ and PO_3_^−^ ions in case of DNA capture probes, which is much less than the case of the MO capture probes as mentioned in the previous section. The distinct difference between the performance of the MO capture probe and the DNA capture probe is also reflected in the intensities of the nucleobase ions (Table S3). Superior performance of the MO capture probe is reflected in 1.4 to 2.3 times more intense peaks in case of MO-based detection compared to DNA-based detection (Fig. 4B). The signal intensities for all four nucleobases were significantly higher for the cases of MO capture probe compared to the DNA capture probe (Welch's *t*-test, *p* < 0.05).

The limit and resolution in DNA quantification can be related to the number of MO/DNA capture probes attached onto the nanoparticles. Since the surface coverage as indicated by the PO_2_^−^ peak intensity is directly proportional to the number of strands attached onto the surface, it is plausible that the higher surface coverage of MO capture probes [as the PO_2_^−^ peak intensity ratio (MO : DNA) is found to be 2.2 : 1 (Fig. 2A and C)] contributes to the stronger DNA hybridization signal. However, surface coverage may not be the only factor, as MO has an intrinsic capacity of forming more stable MO-DNA duplex,^8^ compared to DNA–DNA duplex, due to its structural characteristic like the non-ionic backbone which negates strand–strand repulsion during hybridization. The advantage of morpholino's low electrostatic backbone repulsion has been tested earlier using single molecule force spectroscopy (SMFS) where we have shown superior DNA detection capacity of the MO capture probes, compared to the DNA capture probes.^5,8^ In addition to the mentioned reports,^5,8^ we have recently observed considerably improved DNA detection capacity of the MO capture probes anchored onto AuNPs, compared to the AuNP-anchored DNA capture probes, in a solution-state study using zeta potential-based information (submitted). This work proves that the non-ionic backbone of MO, which offers low electrostatic backbone repulsion, is an advantage of MO for nucleic acid sensing.

**Fig. 5:**
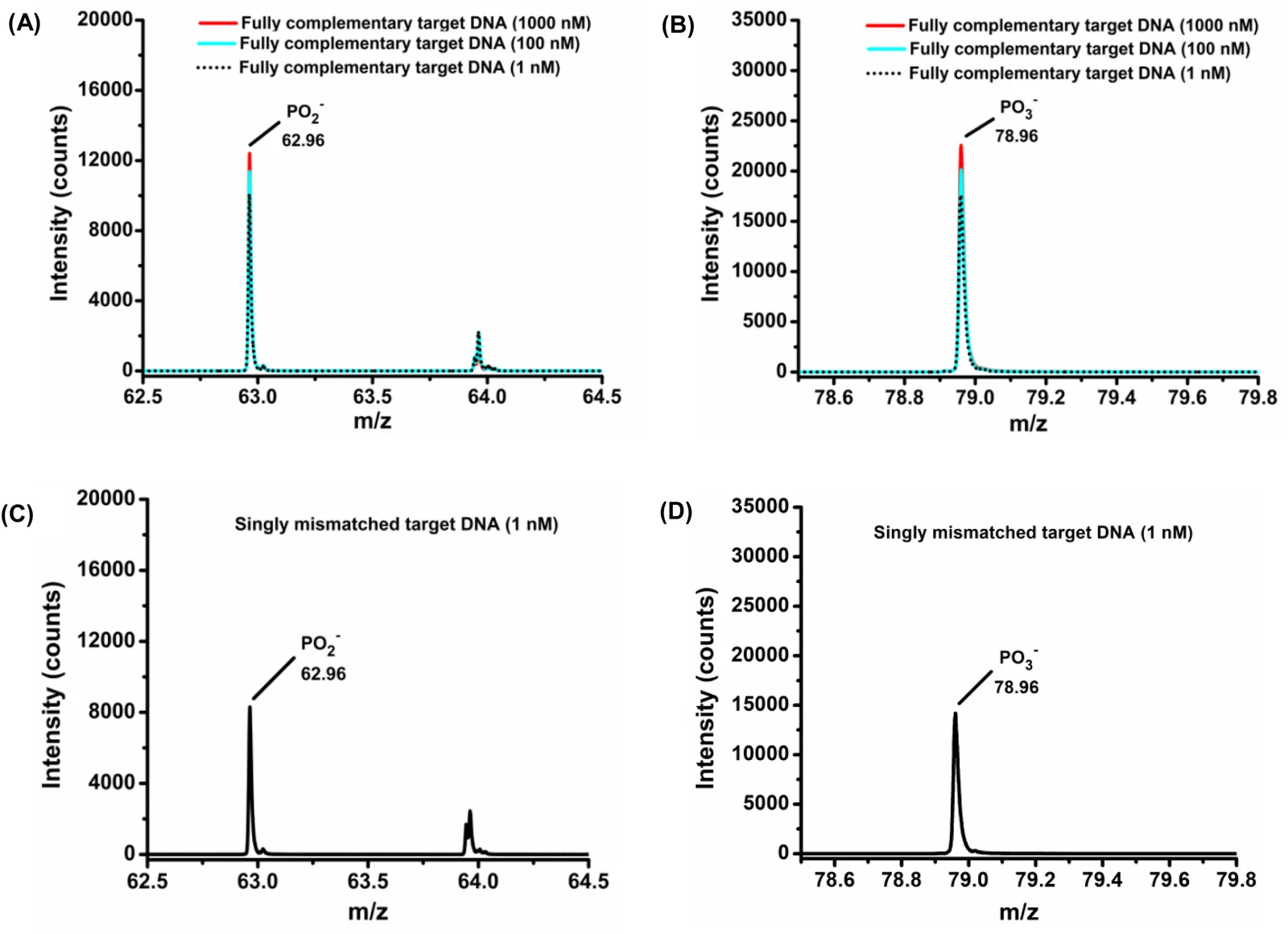
TOF-SIMS negative ion mass peak (*m*/*z*) for (A) PO_2_^−^ and (B) PO_3_^−^ ions represents detection of fully complementary target DNAs of concentrations 1000 nM, 100 nM and 1 nM. In figure (C) and (D) detection of a single base non-complementary target DNA sequence is shown using PO_2_^−^ and PO_3_^−^ ions, respectively.

A concentration dependence study was performed to assess the limit of detection for the MO-based detection of fully complementary target DNA (T-DNA-1_FM_). In Fig. 5A and B, it is shown that the intensities of PO_2_^−^ and PO_3_^−^ decreased gradually with decreasing target DNA concentration from 1000 nM to 1 nM. A singly non-complementary target DNA to HS-MO-1, T-DNA_SMM_ (Table 1) could also be detected successfully (Fig. 5C and D) for target DNA concentration of 1 nM. The gradual decrease in PO_2_^−^ and PO_3_^−^ peak intensities with decreasing target DNA concentration, as seen in Fig. 5, was subjected to quantitative analysis (Fig. S9). Statistical evaluation using pairwise *t*-test confirmed that the intensity differences between the concentrations were significant (*p* < 0.05). The concentration dependence was further quantified by linear regression, which established a statistically significant relationship between the signal intensity and the logarithm of the target DNA concentration for both PO_2_^−^ (*p* = 0.0464, *R*^2^ = 0.9947) and PO_3_^−^ (*p* = 0.0005, *R*^2^ = 0.8448) ions. This robust linear response allowed for the determination of the method's limit of detection. The limit of detection (LOD) was calculated using the formula LOD signal = Mean of blank + 3 × SD of blank, where the blank signal was derived from the PO_3_^−^ intensity of the MO-surface after exposure to non-complementary target DNA (*n* = 7).^24^ This LOD signal was then converted to a concentration using the PO_3_^−^ calibration curve, yielding a value of 1.04 pM. A corresponding calculation based on the PO_2_^−^ signal produced an LOD of 5.49 pM. This demonstrates that the MO-based TOF-SIMS sensing platform can achieve picomolar sensitivity for DNA detection.

### Detection of mutated DNA sequences obtained from non-small cell lung cancer patients

**Fig. 6:**
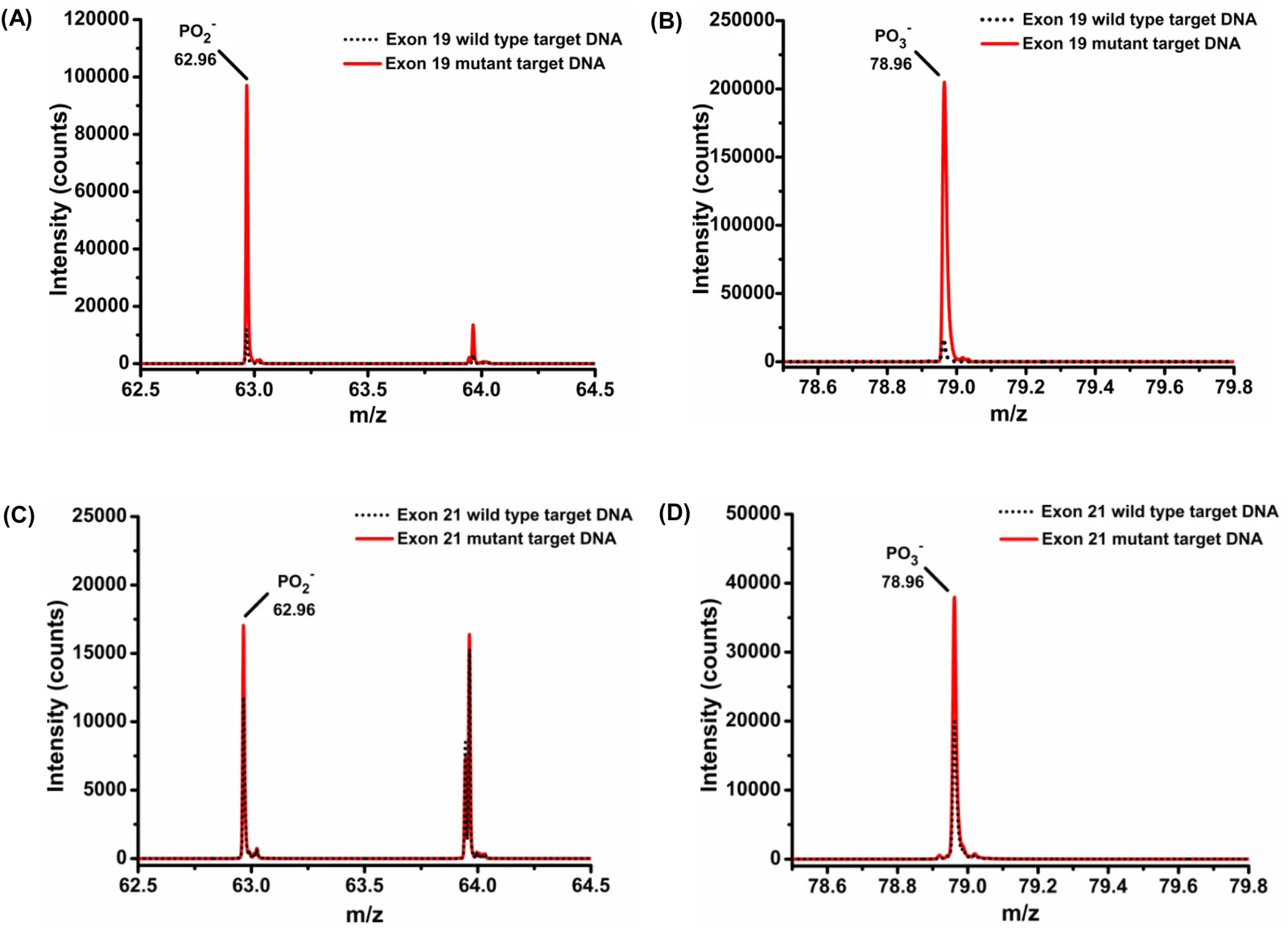
TOF-SIMS negative ion mass peak (*m*/*z*) for (A) PO_2_^−^ and (B) PO_3_^−^ discriminates between exon 19 wild type target DNA and a mutant target DNA. In (C) and (D), discrimination between the exon 21 wild type target DNA and mutant target DNA is shown with PO_2_^−^ and PO_3_^−^ ions, respectively.

After detection of the synthetic fully complementary and singly non-complementary target DNAs using MO capture probe (Fig. 2–5), NSCLC-specific mutated sequences obtained from the CTCs of patient's saliva were addressed for sequence-specific detection using MO capture probe. The sequence having exon 19 deletion mutation (T-DNA_EXON 19 (Mut)_) (Table 1) was fully complementary to the capture probe HS-MO_EXON 19_, and the wild type sequence T-DNA_EXON 19 (WT)_ was non-complementary to HS-MO_EXON 19_ (Table 1). In Fig. 6A and B, it is observed that using both PO_2_^−^ and PO_3_^−^ peaks, clear discrimination could be made between the mutated sequence having exon 19 mutation and the wild type target DNAs, where the discrimination ratios are 8 : 1 for PO_2_^−^ and 10 : 1 for PO_3_^−^. The MO capture probe, HS-MO_EXON 21_, could also discriminate between exon 21 mutant and wild type sequences (T-DNA_EXON 21(Mut)_ and T-DNA_EXON 21 (WT)_, see Table 1) as evident from the intensity differences of PO_2_^−^ and PO_3_^−^ peaks generated from the mutant target DNA (fully complementary) hybridized with MO capture probe, and the wild type target DNA, which contained a single base mismatch to MO, hybridized to the MO capture probe (Fig. 6C and D). So far, the detection limit could be lowered to 1 nM target DNA concentration using a minimum target DNA of volume 2–5 µL (Fig. S10). The clear discrimination between wild-type and mutant sequences, visually apparent in the mass spectra (Fig. 6), was quantitatively validated. Statistical analysis confirmed that the signal intensity for the mutant DNA was significantly higher than for the wild-type DNA for both the exon 19 deletion (*p* < 0.0001) and the exon 21 point mutation (*p* < 0.001), as shown in Fig. S11.

The present SIMS-based method is not comparable with the conventional sensors, for example, electrochemical sensors, in terms of sensitivity, speed and cost. Also, it is a hybridization assay that provides direct chemical verification of a known target sequence binding to the surface-immobilized MO probes, and does not provide sequence information. A comparative analysis with the conventional methods reveals its distinct advantages as it offers a definitive, label-free, and PCR-free nucleic acid sensing approach. Electrochemical sensors, while attractive for speed and cost, typically rely on indirect redox probe-mediated signalling. This introduces analytical vulnerabilities: susceptibility to false positives from unreacted mediators and electroactive contaminants; matrix-dependent electron-transfer variations; and non-specific interactions between reporters and the electrode surface, leading to distorted readouts.^25^ Sensitivity is often compromised by probe-target size mismatches, which attenuate electron-transfer efficiency.^26,27^ Label-free capacitive formats may not provide sufficient specificity for single-nucleotide mismatch discrimination, as changes in bulk dielectric properties are not precisely commensurate with a point mutation.^28^ In contrast, the SIMS-MO platform directly detects specific fragment ions (PO_2_^−^ and PO_3_^−^) generated from the phosphate backbone of the captured target DNA. This detection is independent of redox chemistry, electron transfer kinetics, or probe-target size parity. Especially definitive is the PO_3_^−^ signal, which arises exclusively from the target DNA when using the non-ionic MO probe (which itself contributes no PO_3_^−^ background), providing an unequivocal molecular fingerprint of successful duplex formation.

Fluorescence-based methods are ubiquitous but intrinsically dependent on extrinsic labels, whose performance is governed by variables such as photostability, pH sensitivity, linker chemistry, and labeling efficiency. These dependencies frequently lead to false positives (from unreacted dyes or non-specific association) or false negatives (from poor labeling or photobleaching).^29^ While quantum dots improve photostability, they introduce challenges with aqueous aggregation. FRET-based methods add complexity, as energy transfer efficiency is highly contingent on the local photonic environment, making quantitative interpretation difficult in complex matrices. The SIMS-MO approach circumvents all label-related complications. The readout is based on intrinsic ion fragments sputtered directly from the analyte, eliminating variability from fluorescent tags and the need for costly, multi-step labeling chemistry. The direct PO_2_^−^/PO_3_^−^ signature provides quantitative, calibration-independent evidence of hybridization.

The core innovation of the SIMS-MO platform is its capacity for direct, unambiguous chemical verification of hybridization. Since SIMS-based detection is direct *via* observation of the specific peaks like PO_2_^−^ and PO_3_^−^, the signal processing time can be minimized, unlike in case of electrochemical/nanopore sensing. The detection of the PO_3_^−^ ion, which arises exclusively from the phosphate backbone of target DNA captured by the non-ionic MO probe (which itself contributes no PO_3_^−^ background), provides an unambiguous molecular fingerprint of successful duplex formation. Generation of reliable signal should be more important than achieving ultrahigh sensitivity, high-speed detection and reduction in the cost of detection. The SIMS-MO-based approach combines the intrinsic specificity of mass spectrometry with the benefits of a stable, high-affinity synthetic capture probe, presenting a compelling alternative for applications where reliability, direct molecular evidence, and the avoidance of enzymatic amplification or external labels are prioritized over maximal throughput or minimal cost.

In summary, the SIMS-MO platform synthesizes the strengths of mass spectrometry – direct and label-free molecular identification, with the benefits of a high-affinity, nuclease-resistant synthetic capture probe. It overcomes the key limitations of the major hybridization-based nucleic acid sensing methods, for example, the indirect signalling of electrochemistry and the photophysical variability and label-dependence of fluorescence. While accessibility of a SIMS instrument may be a difficulty, the method presents an attractive alternative for applications where reliability, direct molecular evidence, and avoidance of enzymatic amplification or external labels are priority. The present study demonstrates the applicability of the MO-based SIMS platform for detection of clinically relevant mutations in NSCLC. However, the approach is not limited to this specific condition since target DNA sequences derived from any genetic disease-linked samples can in principle be detected by using suitably designed MO sequences, as the detection strategy relies on sequence-specific hybridization between the MO capture probe and its fully complementary target, regardless of the disease context. In this work, the target DNA lengths applied were as long as 30–40 mer, but we expect that variable lengths of target DNA can be detected, since the MO-based detection relies on the appearance of the PO_3_^−^ mass peak as a universal marker for hybridization. Regarding sample purity, detection in complex matrices containing molecules with non-specific sources of PO_3_^−^ may compromise unambiguous identification of specific target sequences; therefore, sample purification remains important for reliable detection.

## Conclusions

In conclusion, the superior DNA detection ability of the MO capture probes compared to DNA capture probes, under similar preparative and experimental conditions, has been exemplified. It has been shown that the surface-anchored capture probe-AuNP conjugates can be successfully used for DNA sensing using secondary ion mass spectrometry. Discrimination between a fully complementary target DNA and a fully non-complementary target DNA sequence could be achieved by the presence or absence of the PO_3_^−^ ion in negative ion mass spectra. To the best of our knowledge, this is the first report where DNA sequences have been detected using MO-AuNP conjugate system with secondary ion mass spectrometry. The method is simple and is devoid of any fluorescent labelling or end-modification in the target DNA. We are tempted to propose that with application of MO, preparation of the sensing surface becomes simpler than the case of DNA capture probe that usually needs support from materials like PEG/co-spacers for optimal performance. A low sample concentration (1 nM) can be detected, which is the lowest concentration of DNA detected by TOF-SIMS so far. Although the PCR-based methods are sensitive, generally offering comparable limit of detection, and accessibility to SIMS may be limited too due to its high cost, yet SIMS appears to be an attractive alternative to PCR-dependent methods since SIMS-based reading (here, PO_2_^−^, PO_3_^−^ and nucleobase peaks) is devoid of the challenges of PCR, which are contamination and user-incorporated errors during execution of the multi-step process, mispriming, expensive enzymes and false-positive/false negative results. The PO_3_^−^ signature peak intensity alone could serve as the basis for a simple MO-based DNA detection method, provided that SIMS instrumentation becomes more widely accessible. Furthermore, the successful application of Non-negative Matrix Factorization (NMF) for unsupervised spectral decomposition demonstrates that the SIMS-MO platform can be coupled with machine learning tools for data-driven diagnostic analysis. This approach is scalable and can be extended to a wider range of targets and clinical application.

While the present study focused on single-base mismatch discrimination, which is the most stringent test of hybridization specificity, future work will extend this platform to double and multiple mismatches, which are expected to be discriminated with even greater confidence due to increased thermodynamic destabilization. Tests will also be carried out to detect longer target DNA sequences than tested herein. A more detailed analysis relating LOD to probe density and hybridization efficiency will be addressed in future work. Systematic evaluation of storage stability of the MO-AuNP chips will also be addressed in future work. However, given that MO is both nuclease-resistant and protease-resistant, the MO-modified chips are expected to retain activity under storage conditions for longer periods compared to the DNA-modified chips.

In future, an attempt may be taken to achieve detection using the sample collected as is, although working in complex matrices, as is the case of real biological samples (*i.e.*, without purification), could make detection more complex, more ambiguous, and chances of false positive/false negative signals could be higher. In disease diagnostics, the most important requirement should be the reliability in detection. Therefore, a careful balance between attempting detection of the biological sample (as is) and reliability in detection needs to be established.

## Conflicts of interest

The authors declare no conflicts of interest.

## Supplementary Material

RA-OLF-D6RA03286A-s001

## Data Availability

The data supporting this article are available in the main text and the supplementary information (SI). The main text contains the representative TOF-SIMS mass spectra. Supplementary information: TOF-SIMS peak intensity tables for all experimental conditions; calibration curves with linear regression analysis; statistical comparisons of PO_2_^−^ and PO_3_^−^ peak intensities; statistical comparisons of nucleobase peak intensities; concentration-dependent detection of exon 19 mutant DNA; statistical validation of NSCLC mutation detection; TOF-SIMS imaging of Au_3_^−^ distribution; TOF-SIMS spectra from different surfaces; and the full non-negative matrix factorization (NMF) machine learning analysis including rank selection diagnostics, component spectral loadings, activation weights, and the top-scoring peak table. Processed datasets supporting the findings are fully presented within the article and its SI. Raw TOF-SIMS instrument files are available from the corresponding author upon reasonable request. See DOI: https://doi.org/10.1039/d6ra03286a.
