# Supplementary material for: Improved nucleic acid sensing, exemplified using pure target DNA sequences, by MO capture probes compared to DNA capture probes, as revealed from PO2−, PO3− and nucleobase mass peaks
Source: RSC Adv. 2026 Sep 17;16(44):55597–609. doi: 10.1039/d6ra03286a (PMC13584705; doi:10.1039/d6ra03286a)
Supplement: Supplementary file 1 [file RA-016-D6RA03286A-s001.pdf]

Supplementary Information (SI) for RSC Advances.
This journal is © The Royal Society of Chemistry 2026

Supplementary Information

Improved nucleic acid sensing, exemplified using pure target DNA sequences, by morpholino oligonucleotide capture probes, compared to DNA capture probes, as revealed from PO_2^-, PO_3^- and nucleobase mass peaks

Budhaditya Bhattacharya[1], Jayanta Kundu[2], Aratrika Ghosal[1], Asmita Paul[1], Md Qasim[2], Sirsha Dattasarma[1], Tanushree Mana[1†], Subhra Sankar Dutta[3‡], Surajit Sinha[2], Sayan Dey[4], Swati Dasgupta[4] and Rupa Mukhopadhyay[1]*

[1]School of Biological Sciences, Indian Association for the Cultivation of Science, Kolkata-700032, West Bengal, India
[2]School of Applied & Interdisciplinary Sciences, Indian Association for the Cultivation of Science, Kolkata-700032, West Bengal, India
[3]School of Chemical Sciences, Indian Association for the Cultivation of Science, Kolkata-700032, West Bengal, India
[4]CliniMed LifeSciences, Stesalit Tower, E-2-3, GP Block, Sector V, Bidhannagar, Kolkata-700091, West Bengal, India

[†]Present address: Indian Institute of Technology Madras, Chennai-600036, Tamilnadu, India
[‡]Present address: Agilent Technologies India Pvt. Ltd., Kolkata-700091, West Bengal, India

Contents

A. Figures

1. Figure S1. UV-visible LSPR absorbance spectrum for 10 nm AuNP solution

2. Figure S2. HR-TEM images of gold nanoparticles

3. Figure S3. Size distribution of AuNPs from DLS

4. Figure S4. TOF-SIMS spectra showing gold-specific peaks from AuNP-coated surface and AFM image of AuNP-coated surface

5. Figure S5. TOF-SIMS imaging of Au_3^- distribution from AuNP-coated surface

6. Figure S6. TOF-SIMS negative ion mass peaks from different surfaces

7. Figure S7. Statistical comparison of PO_2^- and PO_3^- peak intensities from MO-coated and DNA-coated surfaces before and after exposure to non-complementary and fully matched target DNA

8. Figure S8. Statistical comparison of nucleobase peak intensities demonstrating specific DNA hybridization for MO and DNA capture probes before and after hybridization with fully matched target DNA

9. Figure S9. Statistical analysis of concentration-dependent detection of fully matched target DNA using PO_2^- and PO_3^- signal intensities, including linear regression calibration curves

10. Figure S10. Concentration-dependent detection of exon 19 mutant target DNA with MO capture probe using PO_2^- and PO_3^- intensities, and linear regression analysis

11. Figure S11. Statistical validation of NSCLC mutation detection

B. Tables

1. Table S1. AuNP-specific negative ion peaks in TOF-SIMS spectra with corresponding m/z values, peak assignments, and measured intensities

2. Table S2. Comparison of PO_2^- and PO_3^- peak intensities from MO and DNA capture probe surfaces before and after hybridization with fully matched and fully mismatched target DNA

3. Table S3. Intensity values and statistical comparison of nucleobase peaks from fully matched target DNA hybridized with MO and DNA capture probes

4. Table S4. Background-subtracted PO_2^- and PO_3^- signal intensities attributed specifically to captured target DNA after removal of substrate and probe contributions

C. Machine Learning-Based Spectral Fingerprint Analysis for DNA Hybridization

1. Figure I. NMF rank selection diagnostics: reconstruction error, consensus cophenetic coefficient, and silhouette score across factorization ranks (k = 2–9) for TOF-SIMS spectral data

2. Figure II. Full-range spectral loadings of NMF-derived duplex component

3. Figure III. Component activation weights of NMF Component 3 across experimental groups

4. Table A. Top-scoring peaks in the duplex-associated NMF component

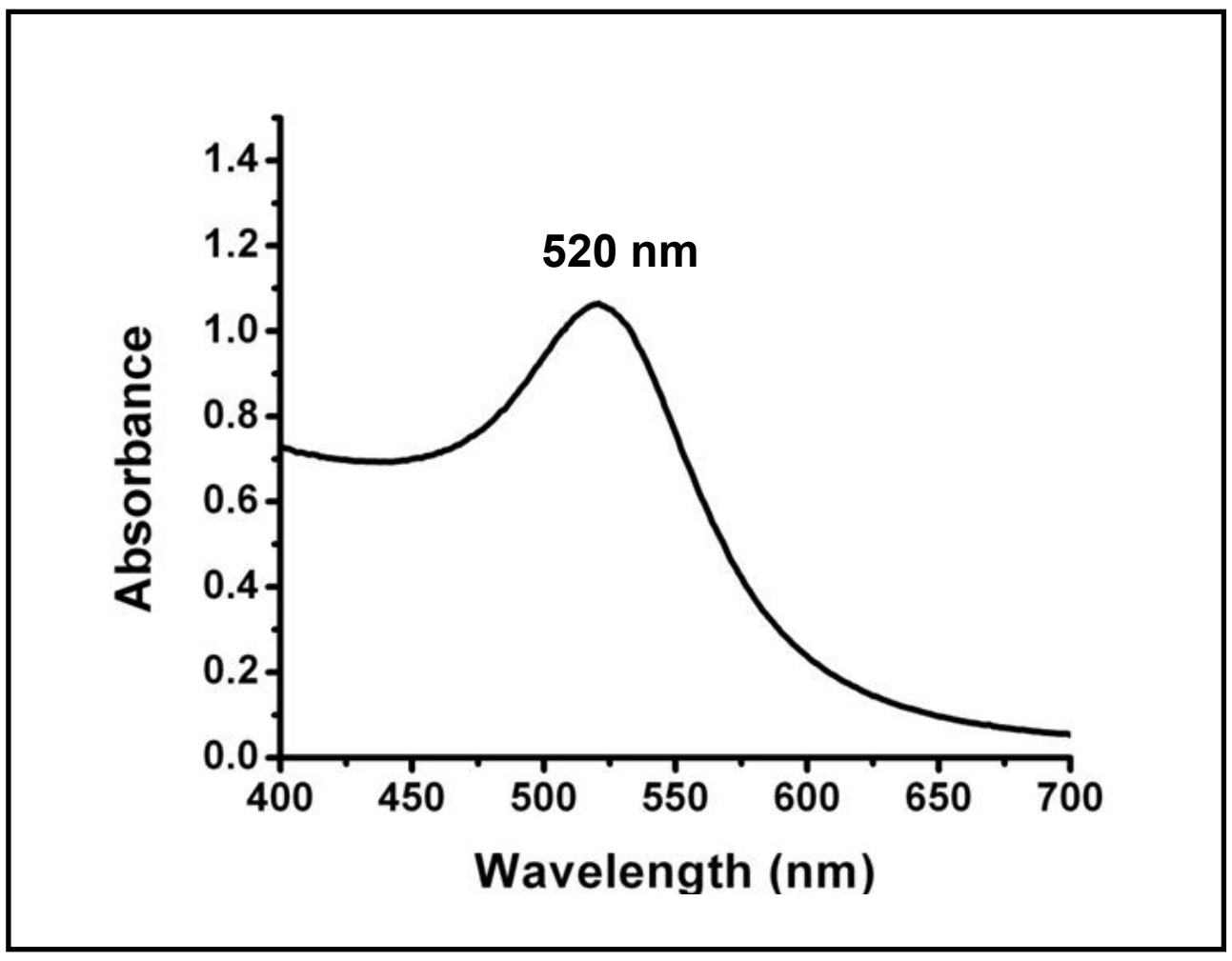

Figure S1. UV-visible LSPR absorbance spectrum for 10 nm AuNP solution. The absorbance peak observed at 520 nm is a characteristic feature of the 10 nm spherical AuNPs.

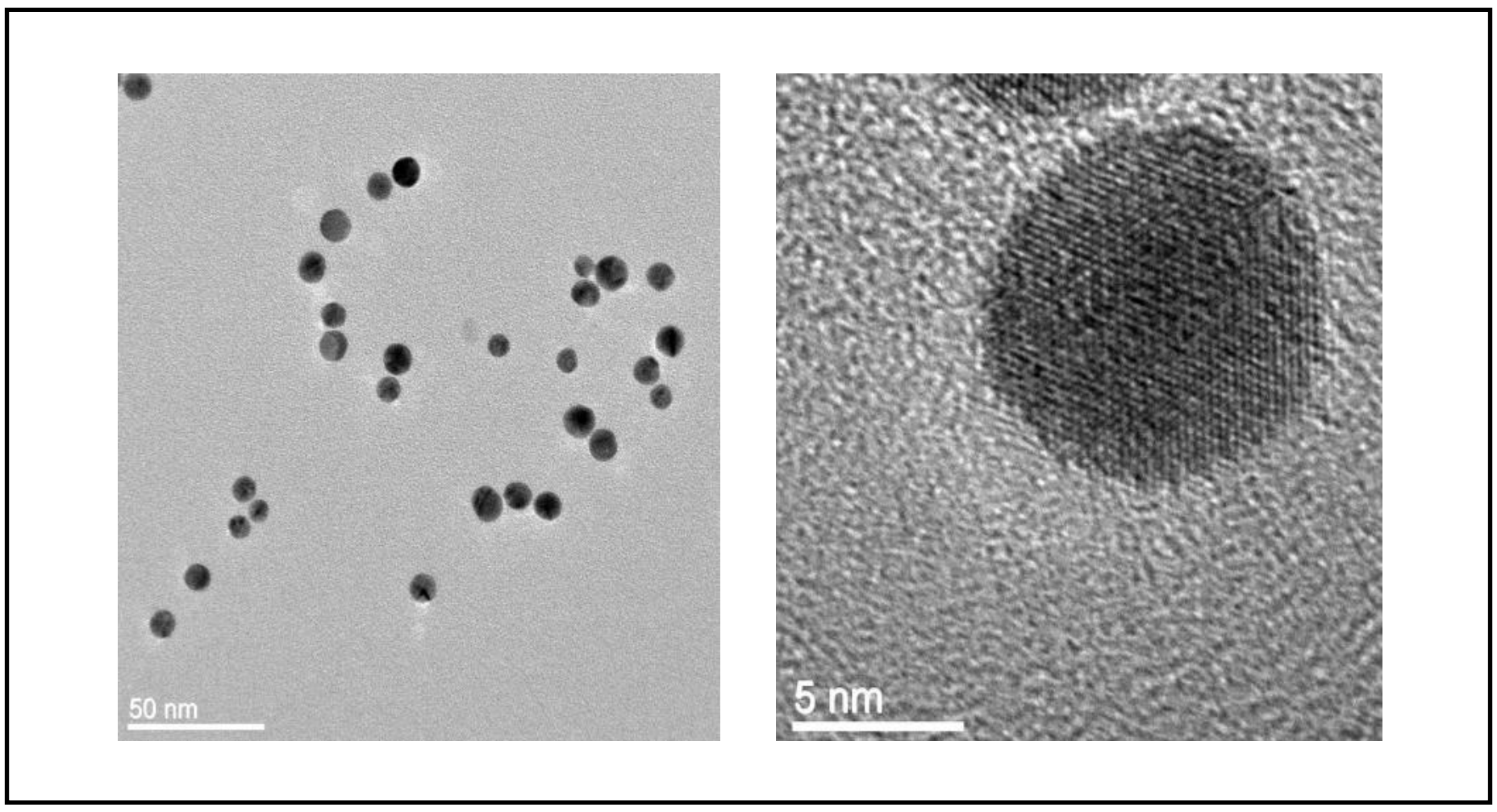

Figure S2. High-resolution transmission electron microscopy (HR-TEM) images of gold nanoparticles (left) and a single nanoparticle (right).

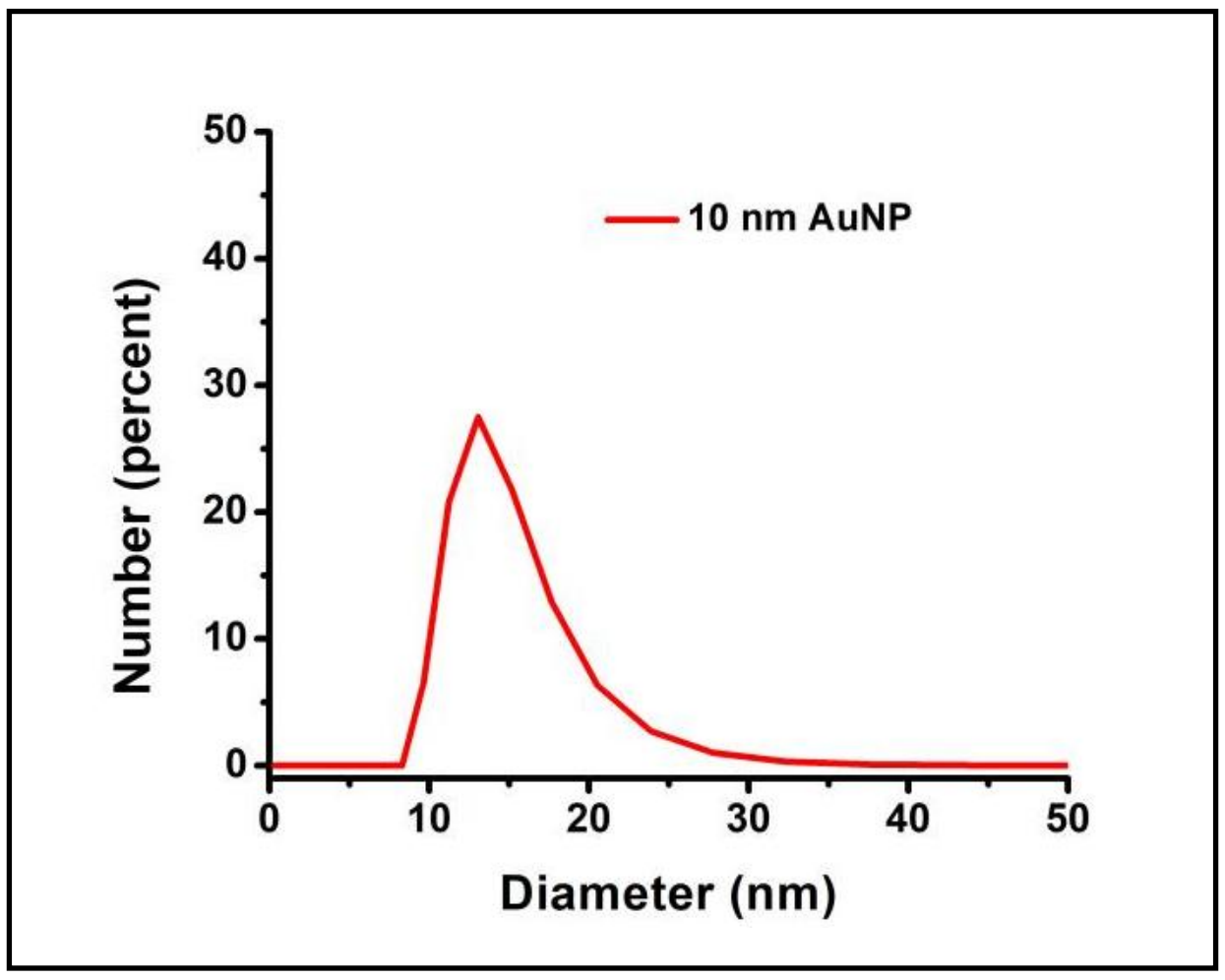

Figure S3. Size distribution of AuNPs obtained from dynamic light scattering experiment. The average diameter was estimated to be 13.88 nm.

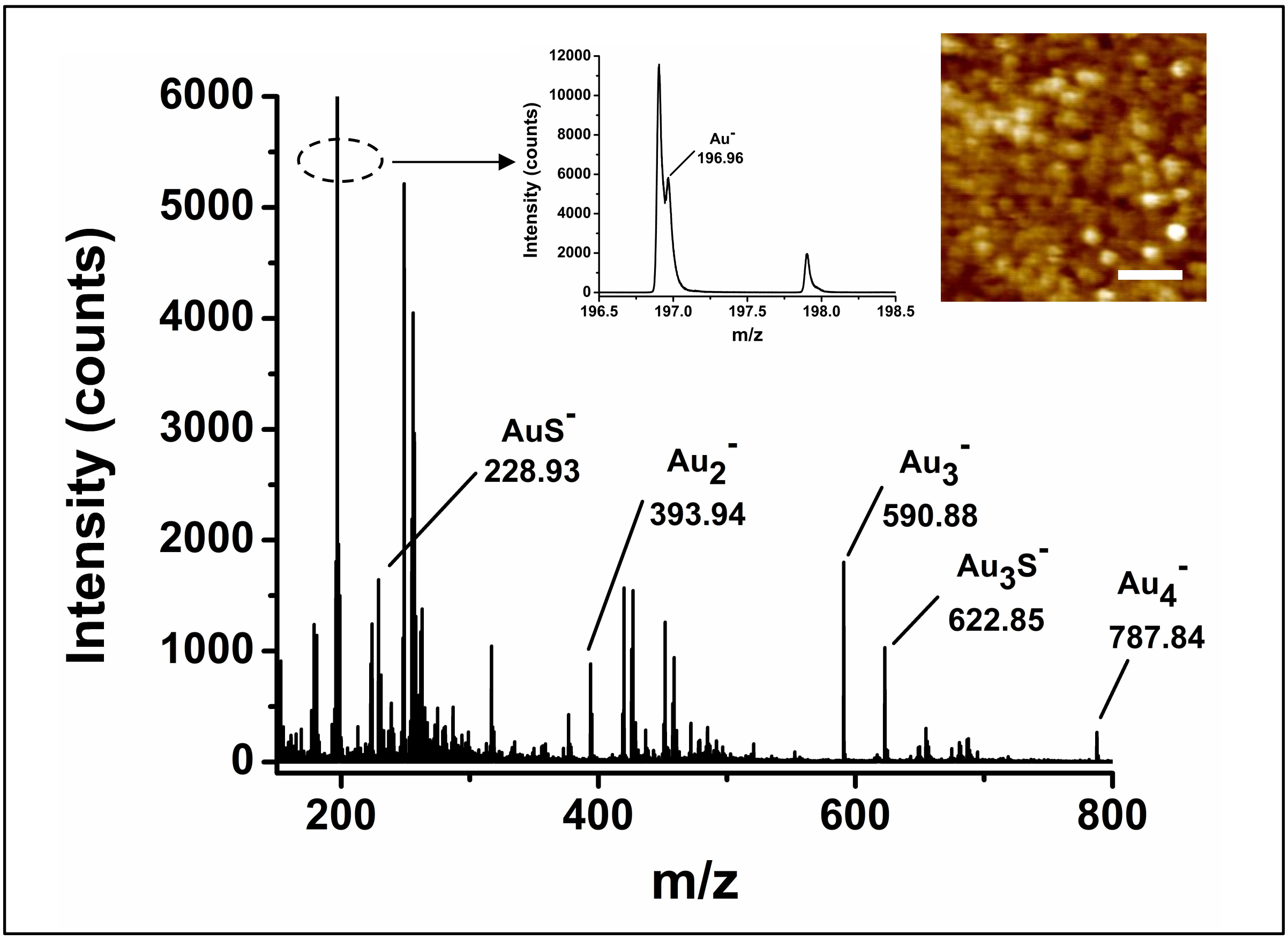

Figure S4. TOF-SIMS spectra in negative ion mode present gold-specific peaks from AuNP-coated glass surface. The peak for Au^- and the AFM image of AuNP-coated surface are shown in the inset. (Scale bar for the AFM image: 300 nm and Z-range: 0-4 nm)

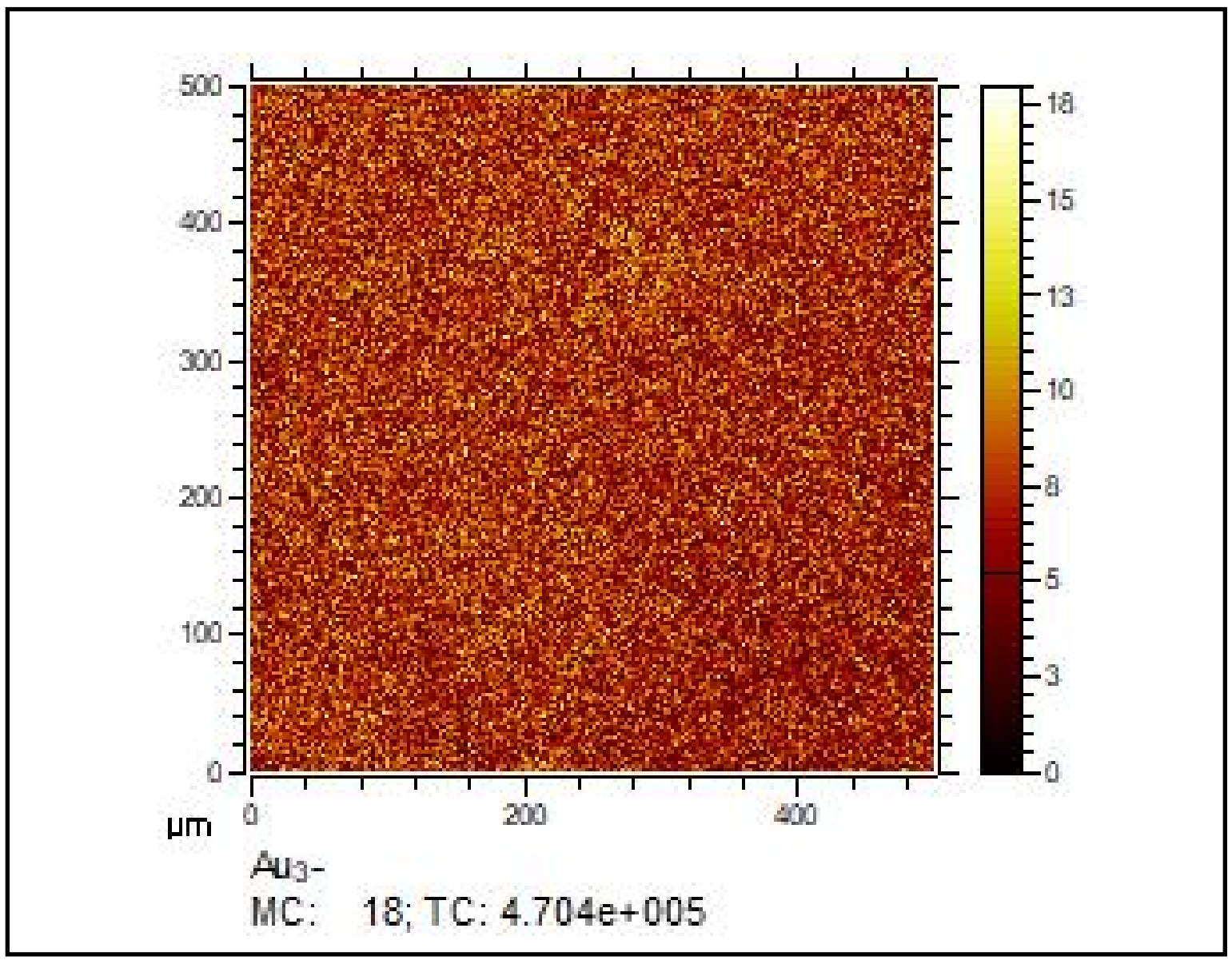

Figure S5. TOF-SIMS imaging using m/z of Au_3^- at 590.88 from a AuNP-coated surface reveals the spatial distribution of AuNPs on the surface.

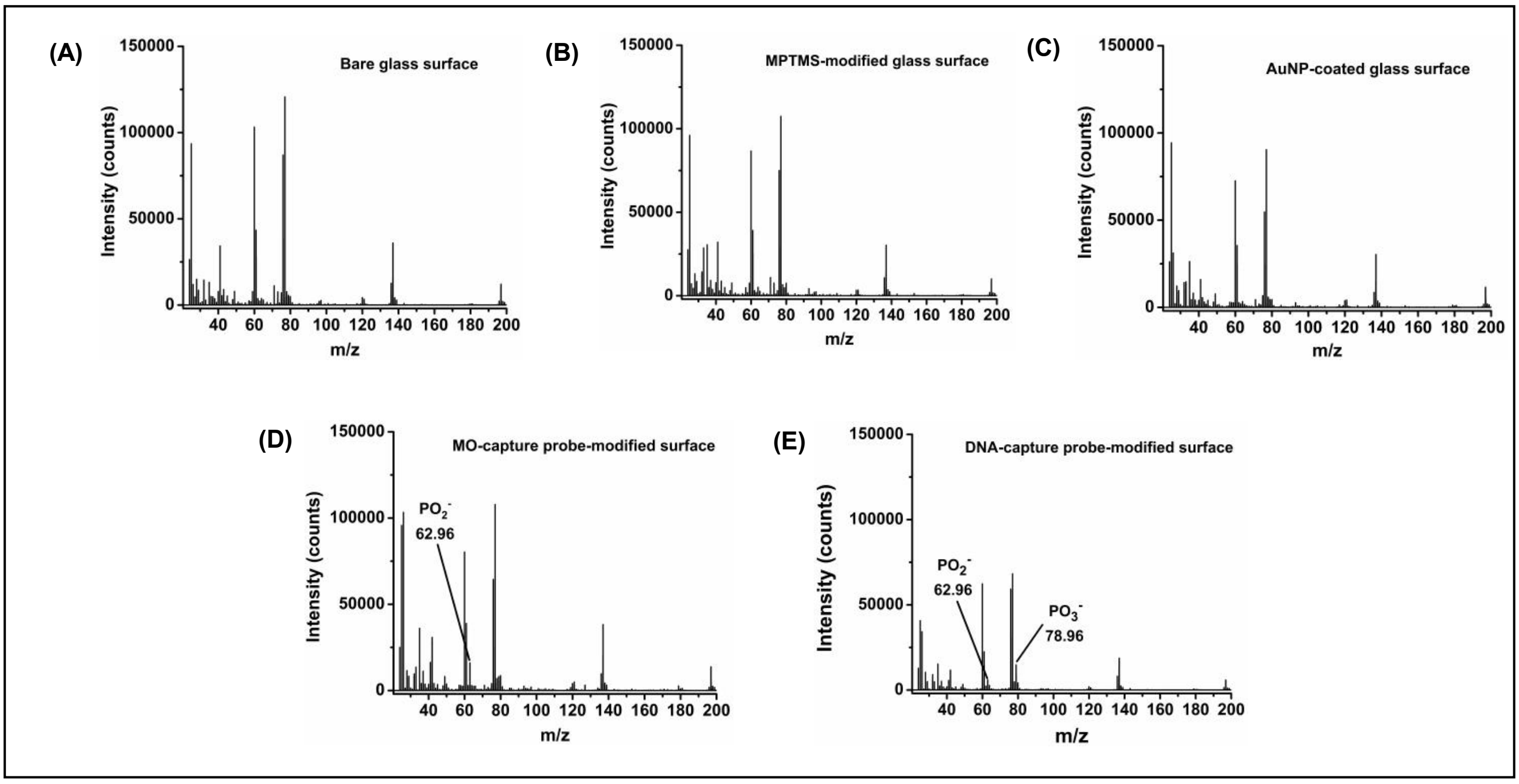

Figure S6. TOF-SIMS negative ion mass peaks from different matrices in the range of m/z 20-200 for (A) bare glass surface, (B) MPTMS-modified glass surface, (C) AuNP-coated surface, (D) MO capture probe-modified surface and (E) DNA capture probe-modified surface. The appearance of only the PO_2^- peak after immobilization of MO is clear from (D), whereas for DNA capture probe-coated surface both PO_2^- and PO_3^- ion peaks are present (E). since both of the ions can arise from the DNA backbone.

.

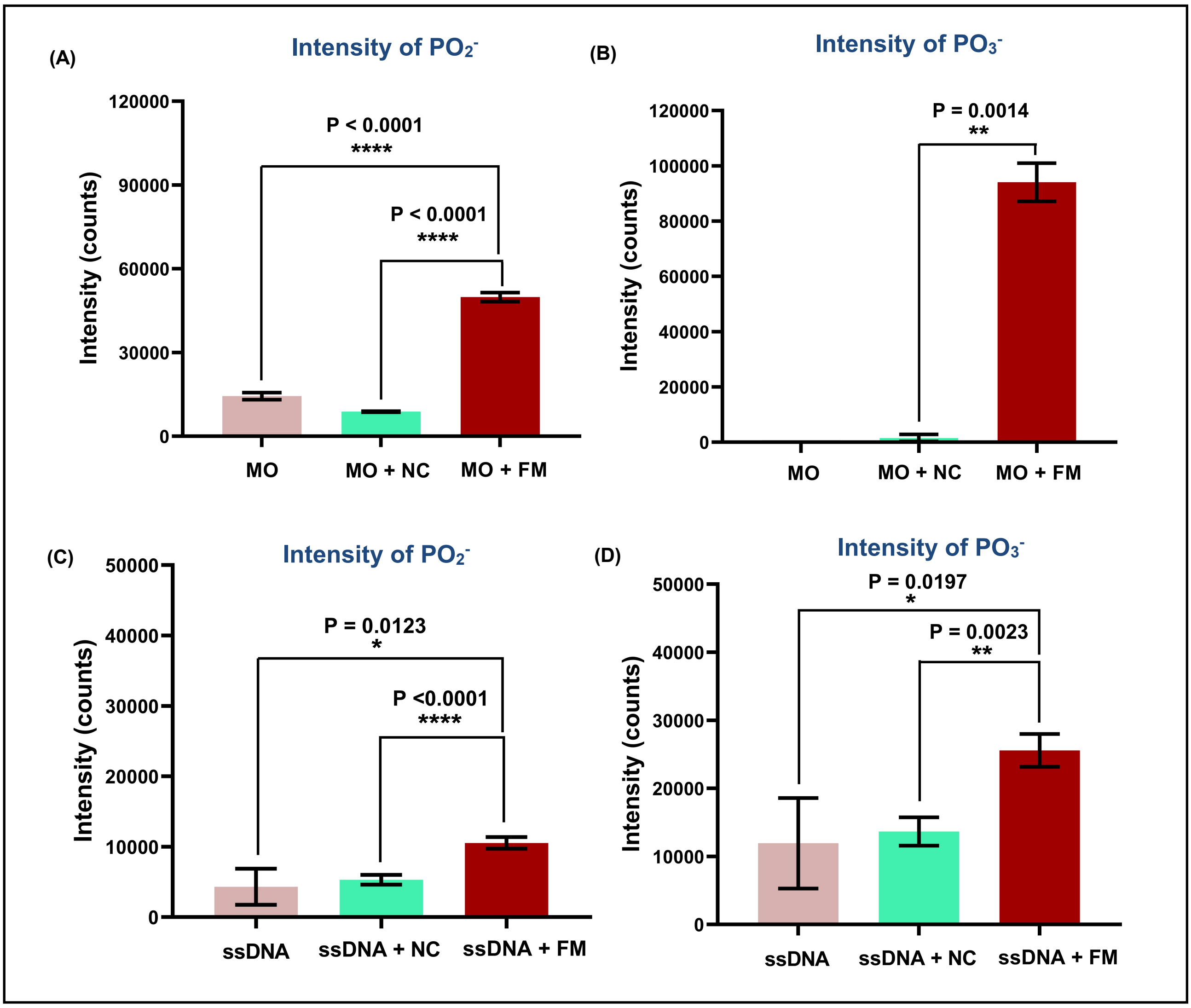

Figure S7. Comparison of TOF-SIMS peak intensities for (A) PO_2^- (m/z 62.96) and (B) PO_3^- (m/z 78.96) ions from the MO-coated surface, and for (C) PO_2^- (m/z 62.96) and (D) PO_3^- (m/z 78.96) ions from the DNA-coated surface under three conditions: as-prepared, after exposure to fully non-complementary target DNA, and after hybridization with fully matched DNA. Error bars represent standard deviation (± SD).

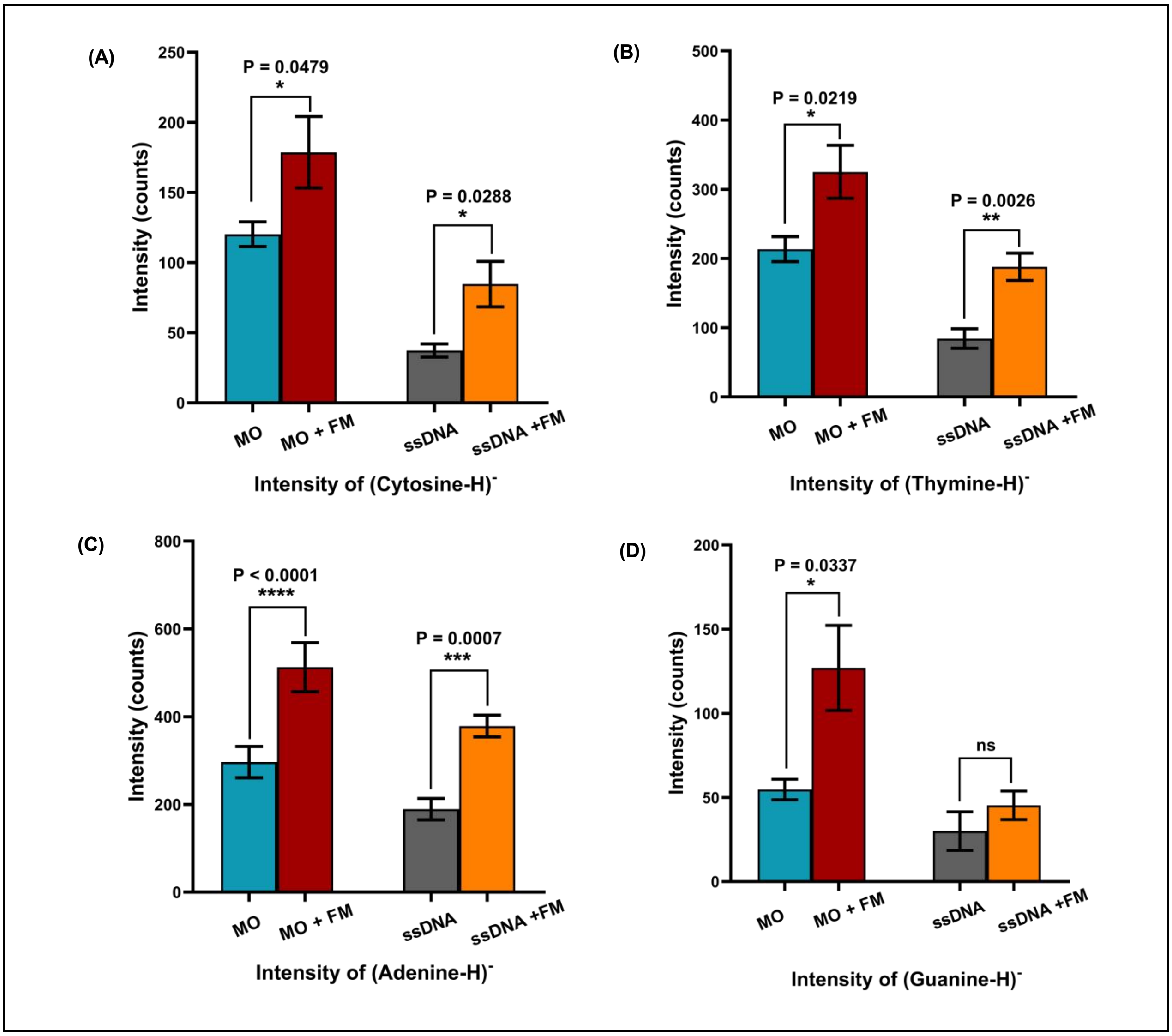

Figure S8: Quantitative comparison of nucleobase peak intensities demonstrating specific DNA hybridization. Bar diagrams show the intensities for (A) (Cytosine-H)⁻, (B) (Thymine-H)⁻, (C) (Adenine-H)⁻, and (D) (Guanine-H)⁻ ions for the MO capture probe and DNA capture probe surfaces, before and after hybridization with the fully matched (FM) target DNA. Error bars represent standard deviation (±SD). For the MO probe, hybridization resulted in a statistically significant signal increase for all four nucleobases (Cytosine: p=0.0479; Thymine: p=0.0219; Adenine: p<0.0001; Guanine: p=0.0337; Welch's t-test). Similarly, for the DNA probe, a significant increase was observed for Cytosine (p=0.0288), Thymine (p=0.0026), and Adenine (p=0.0007), but not for Guanine (ns, not significant).

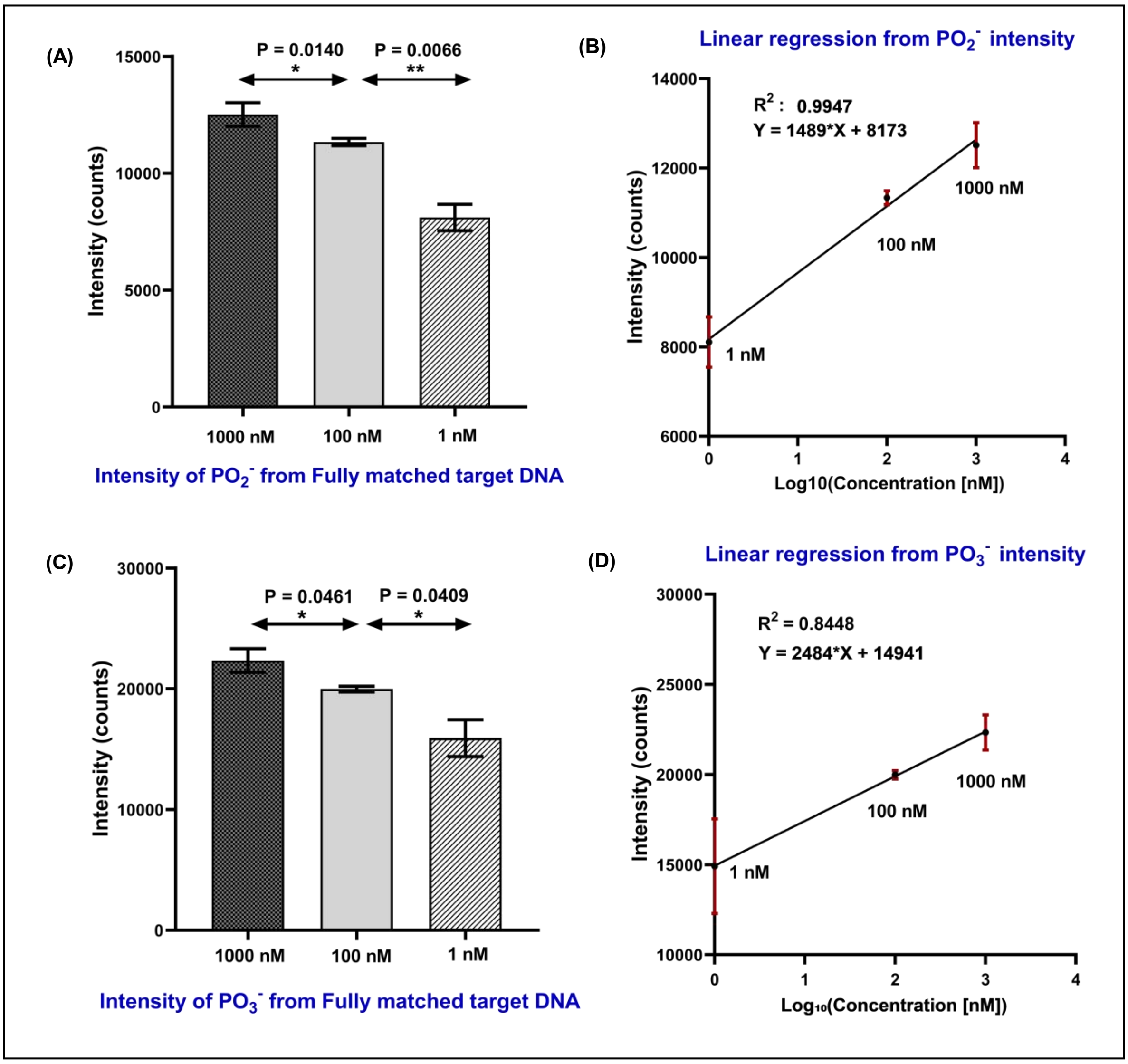

Figure S9. (A) Bar diagram of PO_2^- signal intensities for fully matched target DNA at 1000, 100, and 1 nM. Asterisks indicate statistically significant differences between concentrations as determined by pairwise t-test ($p < 0.05$). (B) Linear regression analysis of the mean PO_2^- intensity from (A) plotted against the logarithm of the target DNA concentration. The solid line represents the best-fit line (Y = 1489*X + 8173, $R^2 = 0.9947$), with a slope that is statistically significantly non-zero (p=0.0464). (C) Bar diagram of PO_3^- signal intensities for the same target concentrations. Asterisks indicate statistically significant differences between concentrations (pairwise t-test, $p < 0.05$). (D) Linear regression analysis of the PO_3^- data from (C) (Y = 2484*X + 14941, $R^2 = 0.8448$), demonstrating a highly significant linear trend (p=0.0005). All error bars represent ±SD. The bar diagram and calibration curve were constructed with n=3 replicates per concentration.

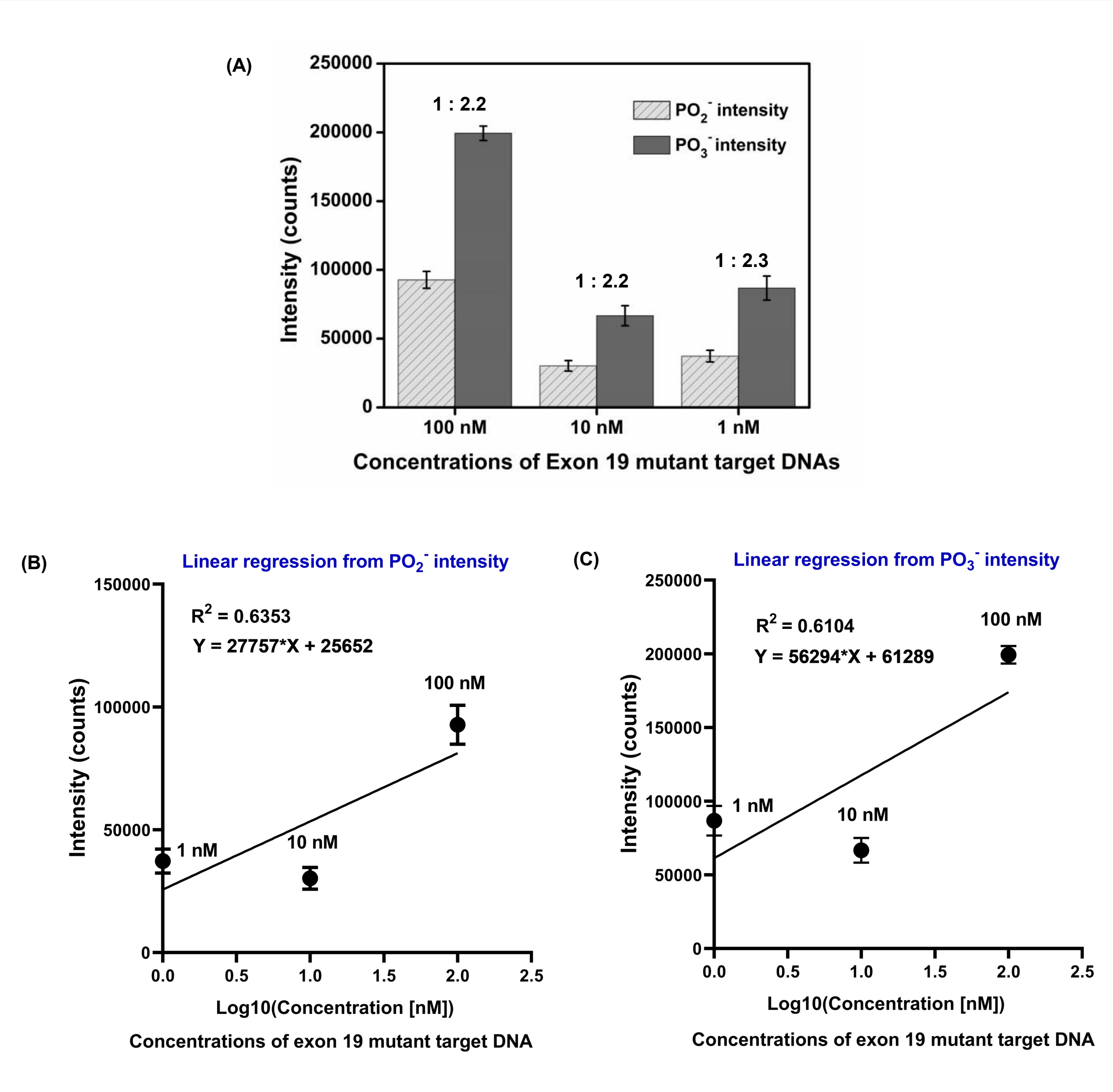

Figure S10. (A) Bar diagram for concentration dependent detection of exon 19 mutant target DNA with MO capture probe using intensities of both PO_2^- and PO_3^-. The PO_2^-: PO_3^- ratio is mentioned for each case. (B), (C) Linear regression analysis for the detection of patient-derived exon 19 mutant DNA. Mean signal intensity is plotted against the logarithm of the target DNA concentration for (B) the PO_2^- ion and (C) the PO_3^- ion. The solid lines represent the best-fit lines, with their equations and coefficients of determination (R^2) displayed on the graphs.

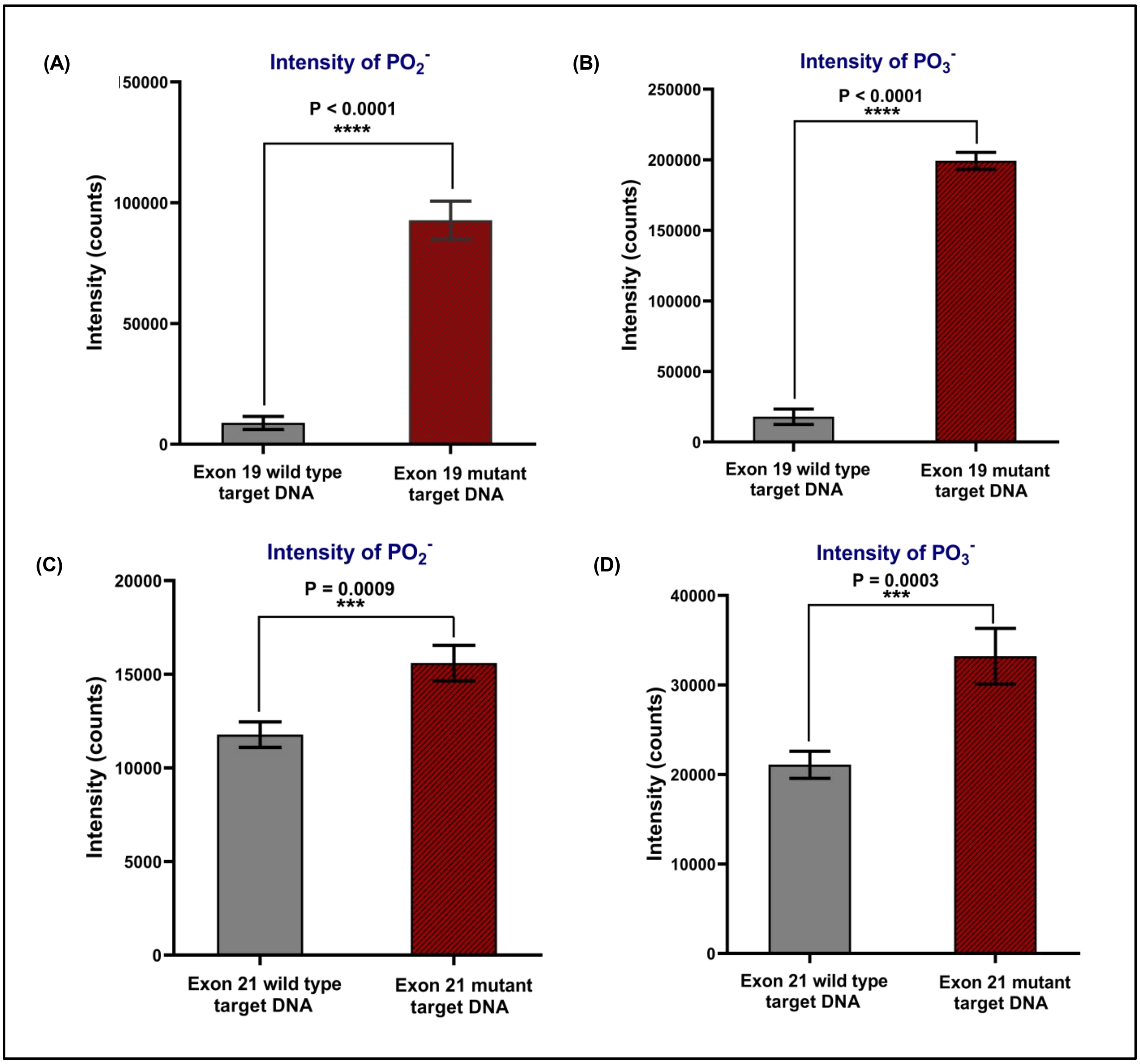

Figure S11: TOF-SIMS analysis for the specific detection of NSCLC mutations. Bar diagrams show peak intensities for (A) PO_2^- (m/z 62.96) and (B) PO_3^- (m/z 78.96) ions from the MO capture probe surface after exposure to the wild-type (WT) and mutant target DNA sequences for the exon 19 deletion mutation. The signal intensity for the mutant DNA is significantly higher than for the wild-type DNA ($p < 0.0001$, Welch's t-test). Bar diagrams for (C) PO_2^- and (D) PO_3^- ions show the same for the exon 21 point mutation, where the signal intensity for the mutant DNA is also significantly higher ($p < 0.001$, Welch's t-test). This demonstrates clear discrimination and specific detection of both mutations. Error bars represent ± SD.

Table S1. AuNP-specific peaks as observed in negative ion mode SIMS spectra

**Peak position (m/z)**	**Peak assignment**	**Peak intensity for AuNP-coated surface**
196.96	Au^-	5745
228.93	AuS^-	1643
393.94	Au_2^-	1092
590.88	Au_3^-	2389
622.85	Au_3S^-	1258
787.84	Au_4^-	353

Table S2. Intensity of peaks for PO_2^- and PO_3^- for MO/DNA-coated surfaces after hybridization with fully matched target DNA and after exposure to fully mismatched DNA. (The values are presented as mean ± standard deviation for n =3)

**Sample type**	**Peak position (m/z)**	**Peak assignment**	**Peak intensity for MO/DNA-coated surface after exposure to fully mismatched target DNA**	**Peak intensity for MO/DNA-coated surface after hybridization with fully matched target DNA**
For MO capture probe case	62.96	PO_2^-	8836 ± 154	49871 ± 1653
	78.96	PO_3^-	1582 ± 1251	94123 ± 6912
For DNA capture probe case	62.96	PO_2^-	5328 ± 692	10550 ± 823
	78.96	PO_3^-	13685 ± 2084	25578 ± 2412

Table S3. Intensity values and statistical comparison of nucleobase peaks from fully matched target DNA hybridized with MO and DNA capture probes. Values are presented as mean ± SD. The p-value indicates the statistical significance of the difference in signal intensity between the MO and DNA capture probes for each nucleobase, as determined by Welch's t-test.

**Nucleobase peak (m/z)**	**Intensity for target DNA fully complementary to MO probe (Intensity 1)**	**Intensity for target DNA fully complementary to DNA probe (Intensity 2)**	**Intensity 1: Intensity 2**	**Statistical significance between Intensity 1 and Intensity 2**
$(Cytosine\text{-}H)^-$ 110.04	178 ± 25.48	85 ± 16.77	2.09	P = 0.0057 **
$(Thymine\text{-}H)^-$ 125.05	305 ± 49.15	187 ± 19.70	1.63	P = 0.0396 *
$(Adenine\text{-}H)^-$ 134.05	511 ± 55.98	379 ± 24.76	1.34	P = 0.0016 **
$(Guanine\text{-}H)^-$ 150.05	127 ± 25.24	55 ± 8.50	2.30	P = 0.0214 *

Table S4. Background-subtracted PO_2^- and PO_3^- signal intensities attributed to captured target DNA, calculated after removing the contribution from the MO or DNA probe layer, used for the analysis in Figure 4A (values presented as mean ± SD).

m/z	Target DNA intensity for the case of MO capture probe ± SD	Target DNA intensity for the case of DNA capture probe ± SD
PO_2^-	35475 ± 1585	6208 ± 1027
PO_3^-	91565 ± 7146	13612 ± 2331

Unsupervised Machine Learning via Non-Negative Matrix Factorization (NMF) Identifies a Multivariate Spectral Fingerprint for DNA Hybridization in TOF-SIMS

Introduction

Negative-ion time-of-flight secondary ion mass spectrometry (TOF-SIMS) provides high chemical sensitivity for surface-bound oligonucleotides, enabling detection of phosphate-bearing fragments generated from nucleic acid backbones. While conventional duplex-identification approaches often rely on monitoring the relative intensities of PO_2^- and PO_3^- fragments, an unbiased, multivariate analysis of the full spectral fingerprint can offer a more robust validation of hybridization specificity. To this end, we applied non-negative matrix factorization (NMF) to the complete spectral dataset (1–1000 Da) to decompose the complex spectral profiles into chemically interpretable components without prior assumption. This data-driven approach allows for the identification of a reproducible, multi-peak spectral signature associated with duplex formation, moving beyond reliance on manual identification of characteristic ions and providing a more comprehensive assessment of hybridization specificity.

Objective

- Detection of higher-mass peaks (if any) that are specifically enriched upon MO–DNA duplex formation.

- Evaluation of matrix effects by comparing MO, NC, and FM spectra to ensure that observed markers do not arise from shared background contributions.

- Application of machine-learning methods capable of extracting robust and interpretable spectral features.

To address these points, non-negative matrix factorization (NMF) was applied as an unsupervised decomposition method to the full negative-ion spectra. NMF provides a parts-based representation in which each component corresponds to a non-negative spectral pattern and each sample is expressed as a non-negative activation weight. This framework enables

identification of a duplex-associated spectral pattern, quantification of its contribution across experimental groups (MO, NC, FM), and objective evaluation of whether the detected signature arises from specific hybridization or shared background.

Experimental Data and Pre-processing

Raw Spectra

The dataset consists of nine negative-ion TOF-SIMS spectra acquired from three experimental conditions: MO-only (MO), MO with a fully non-complementary target DNA (NC), and MO with a fully matched target DNA (FM), with three replicates per group. All spectra were collected in the 1–1000 m/z window. Raw intensity values were constrained to non-negative values and subjected to total-ion-count (TIC) normalization to account for variation in overall ion yield across replicates. These steps ensure that each spectrum reflects relative abundance differences across m/z rather than differences in total ion flux.

m/z Alignment

The raw spectra differed slightly in their native m/z sampling grids. To enable direct, point-wise comparison across samples, a reference spectrum was chosen as the one containing the largest number of native m/z points. All other spectra were linearly interpolated onto this reference grid, using zero-padding outside the minimal and maximal m/z bounds and re-normalizing post interpolation to maintain TIC consistency. This procedure preserves the high-resolution structure of the original spectra while ensuring that all samples share an identical m/z axis.

Aligned Matrix

After interpolation and normalization, the aligned spectral matrix has the dimension:

$$X_{\text{aligned}} \in \mathbb{R}_{\geq 0}^{9\times 954,778},$$

where each row corresponds to a single sample and each column corresponds to a specific m/z bin on the shared grid. This matrix, saved as X_aligned.npy, forms the input for subsequent NMF-based decomposition and peak-selection analyses.

NMF rank selection

To determine the optimal factorization rank for the full-range spectral data, non-negative matrix factorization (NMF) was evaluated for ranks k ∈ {2, 3…,9}. For each rank, twelve

independent NMF runs were performed using random initialization, and three quantitative diagnostics were computed:

- **Reconstruction error** (mean across runs), assessing the fidelity of the approximation X≈WH;
- **Consensus cophenetic coefficient**, measuring the stability of sample clustering across repeated factorizations at the same rank; and
- **Silhouette score** calculated from the W-based cluster assignments, quantifying the separation between experimental groups (MO, NC, FM).

As shown in Figure I, the reconstruction error decreased marginally beyond k=3, while the cophenetic coefficient exhibited a local maximum at k=3, indicating highly stable and reproducible components. Silhouette scores were also maximized near k=3, consistent with the presence of three distinct experimental groups. Based on the combined criteria of stability, interpretability, and parsimony, k=3 was selected for all subsequent analyses. Higher ranks introduced components with limited reproducibility and without chemically meaningful spectral profiles.

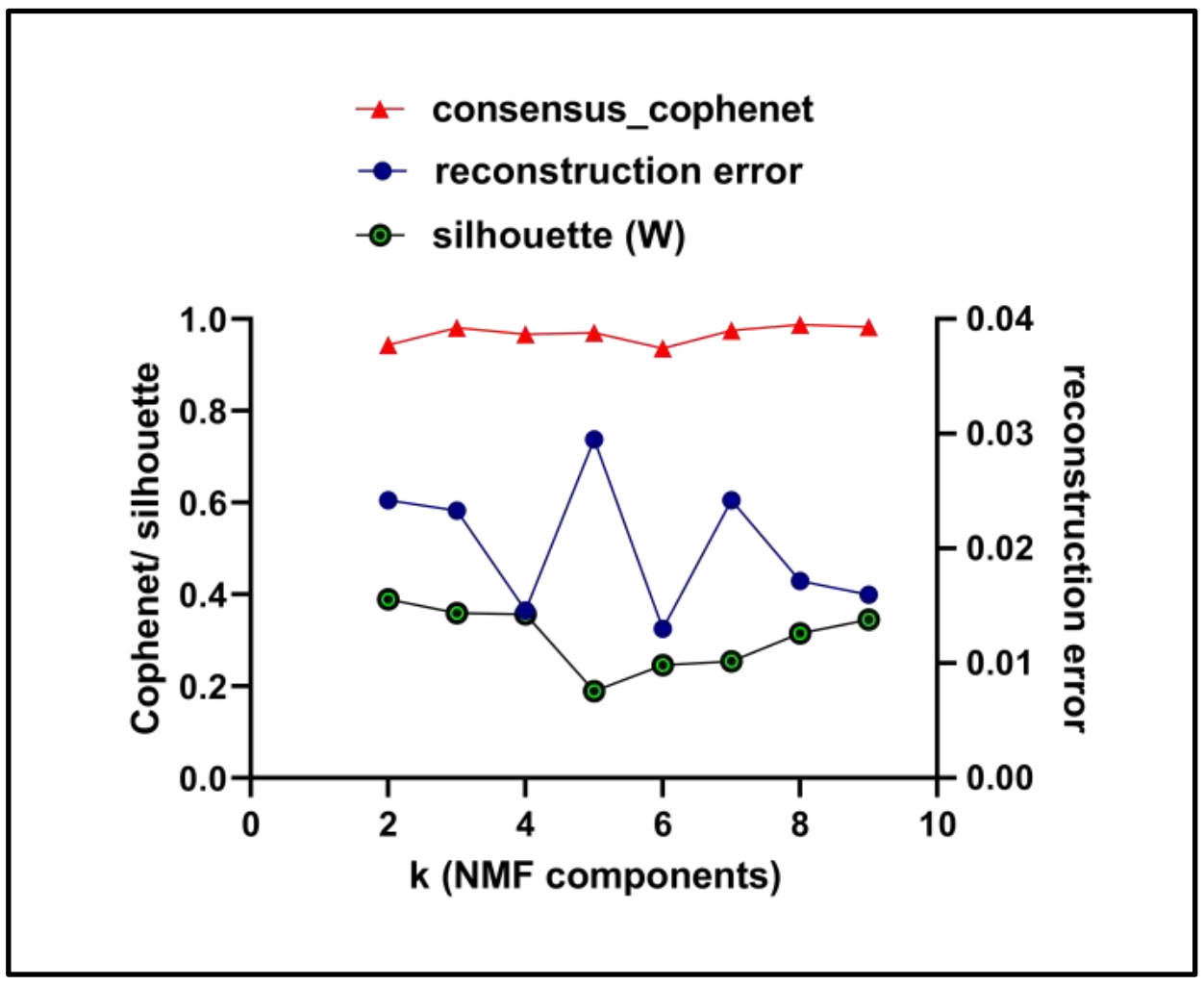

Figure I. NMF rank-selection diagnostics. Reconstruction error shows marginal improvement beyond $k = 3$, whereas the consensus cophenetic coefficient reaches a local maximum near $k = 3$, indicating highly stable solutions. Silhouette scores remain consistent with the presence of three underlying experimental groups (MO, NC, FM).

NMF Decomposition (k=3)

Components and Activations

Applying NMF with $k = 3$ yields a parts-based decomposition of the aligned spectral matrix:

$$X \approx WH,$$

where each row of W encodes the contribution of each component to a given sample, and each row of H represents a non-negative spectral pattern spanning the full 1–1000 Da m/z range (Figure II). For the present dataset,

- $W \in \mathbb{R}_{\geq 0}^{9\times 3}$ contains the component activations for the nine spectra (MO, NC, FM replicates), and
- $H \in \mathbb{R}_{\geq 0}^{3\times 954{,}778}$ contains the three learned component spectra sampled on the common m/z grid.

The non-negativity constraints ensure that each spectral component corresponds to an additive, chemically interpretable signature rather than a mixture of positive and negative basis functions.

Identification of the Duplex Component Using Unsupervised Machine Learning

The three components derived from NMF were evaluated for their relevance to DNA hybridization. Component 3 was unambiguously identified as the duplex-associated signature through a data-driven, unsupervised approach based on two objective criteria:

Spectral Profile: Component 3's spectral loadings are dominated by peaks corresponding to PO_3^- (m/z 78.96) and PO_2^- (m/z 62.96), key fragment ions known to originate from nucleic acid phosphate backbones. The NMF algorithm independently identified these ions as the most significant spectral features distinguishing hybridization events.

Activation Pattern: The component activation weights in W were markedly and exclusively elevated in fully matched (FM) samples, with minimal contributions in MO-only and non-complementary (NC) controls. This demonstrates that Component 3 captures spectral variance specific to successful duplex formation, not matrix or non-specific background.

Thus, without prior bias, NMF isolated a reproducible, phosphate-based spectral fingerprint that serves as a robust, multivariate marker for DNA hybridization.

Table of Top Peaks

Table A lists the highest-scoring peaks extracted from the duplex component (Component 3) using the greedy tolerant-peak selection algorithm (K = 50, ±1 Da exclusion). Scores correspond to component intensities in H and reflect the relative contribution of each m/z value to the duplex associated spectral pattern.

Table A. Top-scoring peaks in the duplex NMF component (Component 3)

m/z	Score	Assignment / Notes
1.01	0.0555	H^- (from surface)
15.99	0.0279	O–
78.96	0.0156	PO_3^- (DNA hybridization-specific)
17.00	0.0153	OH^-
76.97	0.00743	From glass surface
62.96	0.00733	PO_2^- (DNA hybridization-specific)
26.00	0.00596	CN^-
75.96	0.00497	From glass surface
59.97	0.00430	From glass surface
34.97	0.00350	From glass surface
136.94	0.00282	From glass surface

The NMF-derived duplex component is characterized by a clear spectral pattern in which PO_3^- and PO_2^- are the only target-specific peaks among the top contributors. All other high-scoring ions originate from the glass substrate, confirming that the hybridization signature is defined by the specific enhancement of phosphate fragments against a constant, non-interfering matrix. This result directly addresses concerns about matrix effects and validates

the use of phosphate ions as robust hybridization markers. The duplex-associated NMF component (Component 3) includes not only the hybridization-specific phosphate ions PO_3^- and PO_2^-, but also several background peaks originating from the glass substrate (e.g., H^-, O^-, OH^-). Importantly, the absolute intensities of these background ions remain constant across all experimental conditions (MO, NC, FM). Their presence in Component 3 reflects the covariant spectral structure captured by NMF: in FM samples, the significant increase in phosphate signal occurs against a stable, unchanging background. Thus, Component 3 represents the characteristic spectral profile of the hybridized state, defined by elevated PO_3^-/PO_2^- superimposed on a consistent substrate-derived background, rather than an increase in background signal itself. This result confirms that the hybridization signature is specific and matrix-independent, as the background contribution remains invariant across all samples.

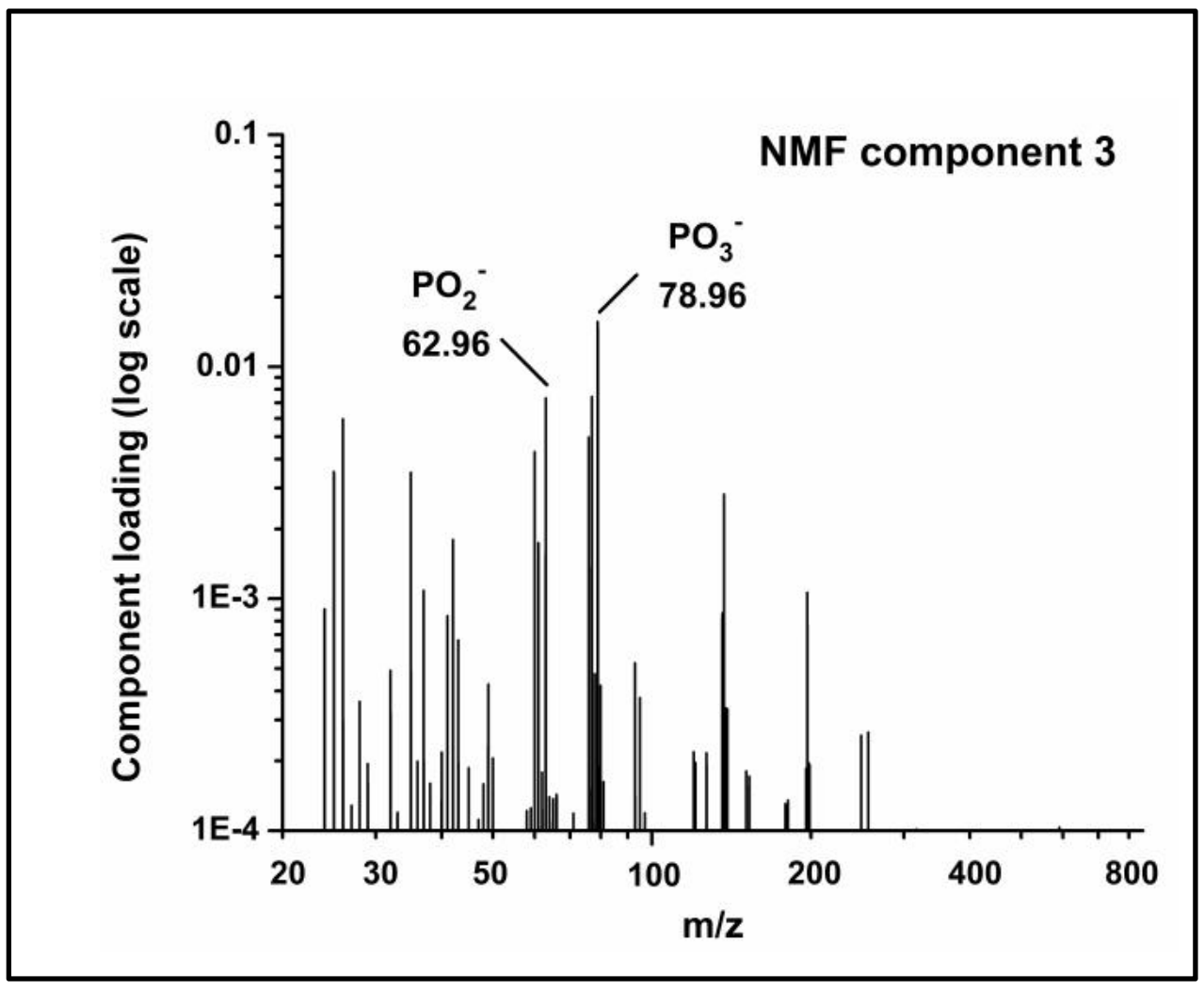

Figure II. Spectral loadings of the NMF-derived duplex component (Component 3).
Full-range view (m/z 20-850, log scale) of Component 3, highlighting the dominant phosphate backbone fragments PO_2^- (62.96 Da) and PO_3^- (78.96 Da)**.**

Component Weights (W) and Statistical Analysis

Group-wise Weights

The activation weights of Component 3, quantifying its contribution to each sample's spectrum, show a clear and specific pattern across experimental groups (Figure III). The mean weight for fully matched (FM) samples (0.0663±0.0315) was 5.6-fold higher than for MO-only controls (0.0119±0.0030) and 15-fold higher than for non-complementary (NC)

DNA controls (0.0044±0.0055). All three FM replicates exhibited higher weights than any MO or NC replicate, demonstrating perfect group separation.

These results confirm that Component 3 captures spectral variance exclusively associated with specific DNA hybridization, with minimal contribution from the capture-probe layer (MO-only) or non-specific DNA adsorption (NC).

Effect Sizes

To quantify the magnitude of group separation independently of sample size, Cohen's *d* was computed for both contrasts:

$$d_{FM–MO} = 2.44,$$

$$d_{FM–NC} = 2.74.$$

Both values correspond to extremely large effect sizes, confirming that the duplex component extracted by NMF provides a robust discriminator between FM and control spectra even when formal hypothesis-testing power is limited by replicate count.

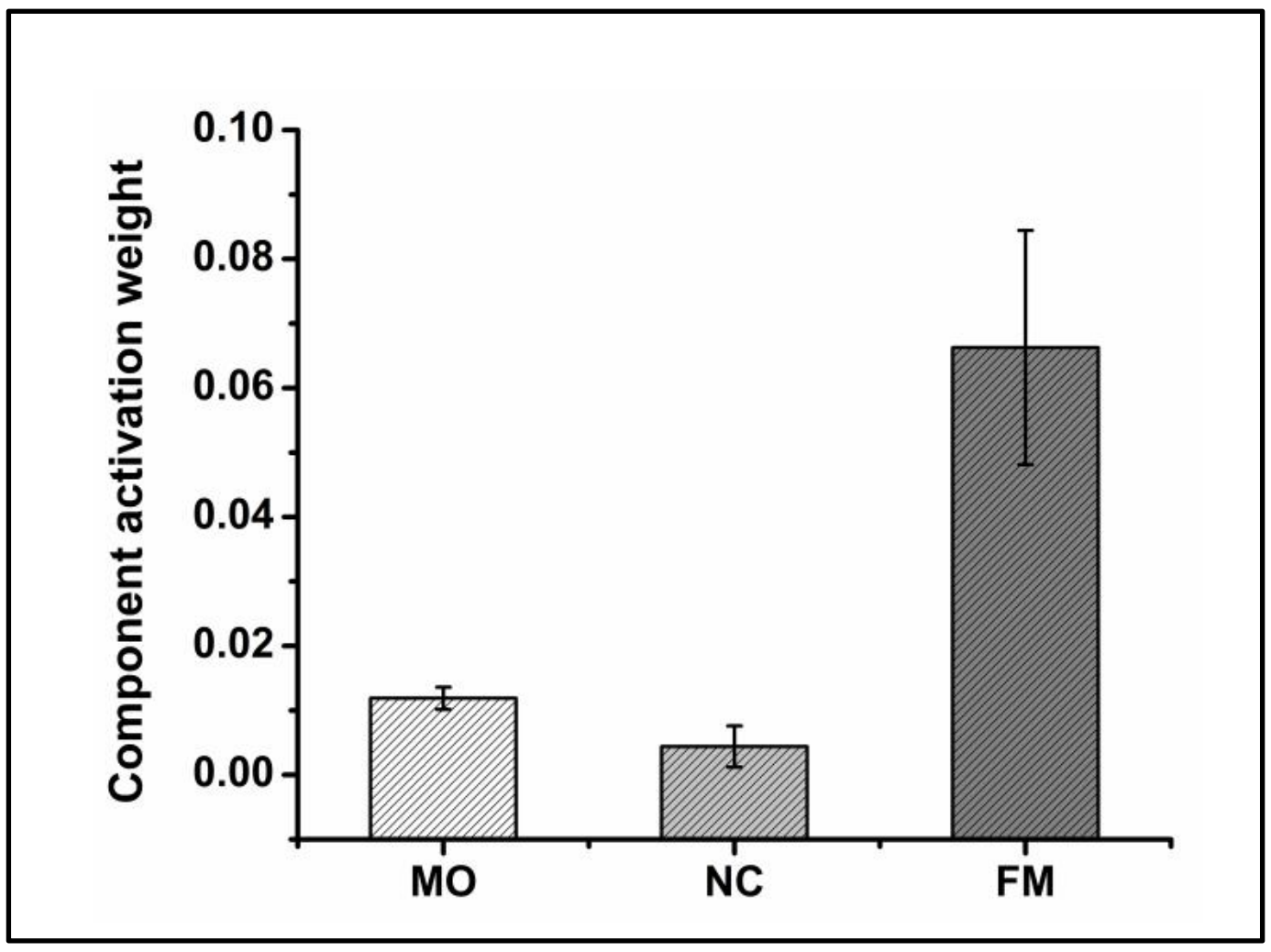

Figure III. Activation weights of NMF Component 3 for MO-only non-complementary DNA control (NC), and fully matched target DNA (FM) conditions.
Component activation weights quantify the contribution of Component 3 to each sample's spectrum. The significant elevation in FM samples demonstrates that this component specifically captures spectral changes associated with target hybridization. Error bars represent standard deviation (n = 3). All FM replicates exceed all MO and NC values.

spectral dataset provided an unbiased, machine-learning-driven validation of our DNA sensing approach. NMF independently identified a single component (Component 3) whose

spectral profile is dominated by the phosphate backbone fragments PO_3^- and PO_2^-, the same ions utilized in our direct detection strategy. This component's activation was 5.6- to 15-fold higher in fully matched (FM) target samples than in MO-only or non-complementary (NC) controls, with all FM replicates exceeding all control values. Large effect sizes (Cohen's $d > 2.4$) further confirm the robustness of this discrimination.

Critically, the constant intensity of background peaks (e.g., glass substrate fragments) across all conditions demonstrates that the observed signature arises specifically from hybridization, not from matrix effects or non-specific adsorption. No higher-mass, sequence-specific fragments contributed significantly to the component, underscoring that PO_3^- and PO_2^- serve as the most reliable and specific spectral markers for DNA hybridization in this TOF-SIMS-based assay.

Thus, the NMF analysis directly addresses our initial objectives by:

- Employing unsupervised machine learning to extract a reproducible spectral fingerprint,

- Confirming the specificity of the phosphate-ion signal to target hybridization,

- Establishing the absence of significant matrix interference, and

- Validating the chosen detection ions (PO_3^-/PO_2^-) as analytically robust for label-free, PCR-free DNA sensing.
